# Estimating daily ozone levels in urban ambient air

**DOI:** 10.1007/s10661-026-15566-w

**Published:** 2026-06-19

**Authors:** David Galán-Madruga, Jafet Cárdenas-Escudero, Parya Broomandi, Javier Lucas Urraca, Jorge Omar Cáceres

**Affiliations:** 1 National Reference Laboratory of Air Quality, National Centre for Environmental Health (CNSA), Carlos III Health Institute (ISCIII), Ctra. Majadahonda a Pozuelo, Madrid, 28222 Spain; 2 Laser Chemistry Research Group, Department of Analytical Chemistry, Faculty of Chemistry, Complutense University of Madrid, Plaza de Ciencias 1, Madrid, 28040 Spain; 3 Analytical Chemistry Department, FCNET, University of Panama, University Mail, 3366, University City, Panama City, Panama 4 Panama; 4 Department of Civil and Environmental Engineering, School of Engineering and Digital Sciences, Nazarbayev University, Nursultan (Astana), Kazakhstan; 5 FCNET, Universidad de Panamá, Panama City, Panama

**Keywords:** Environmental sciences, Air quality, Urban daily ozone levels, Temporal validation, Geographical applicability

## Abstract

Since tropospheric ozone poses a significant threat to public health, this research aims to estimate urban daily ozone levels using 2004–2021 polluting and meteorological data of three major European capitals (Madrid, Stockholm, and Rome). The resulting expression showed a goodness-of-fit of 84.99% (range 2–120 µg O_3_/m^3^), with an average accuracy of 0.01 µg O_3_/m^3^ (equivalent to 0.86%). According to the European legislative requirements for short-term ozone modeling (associated uncertainty < 50%), the total number of 2004–2021 daily exceedances was 0.079% (21 cases). As a novelty, the current work evaluated the performance of the suggested approach at both temporal (using data not included in the original approach) and geographical scales (applied to an area different from those used in the original approach), differing from previous studies that did not address these features. Temporally, a global *r* value of 0.863 was observed, also complying with the current legislative requirements for short-term ozone modeling. The RMSE, MAE, and MAPE metrics were 1.05 μg/m^3^, 1.11 μg/m^3^, and 11.16%, respectively (range 7 to 139 µg/m^3^). Geographically, an *r* value of 0.921 was reached, meeting legislative requirements in 98.8% of data. RSME, MAE, and MAPE values of 1.79 µg/m^3^, 3.20 µg/m^3^, and 12.80% were observed. As a potential limitation, reanalysis datasets were used instead of independent observational data. Despite this, a validated, easy-to-implement approach for estimating daily urban ozone levels using satellite data is provided, reducing uncertainty due to limited information, which could complement European Legislative directives on short-term ambient air ozone modeling.

**Supplementary information:**

The online version contains supplementary material available at 10.1007/s10661-026-15566-w.

## Introduction

Employing modeling techniques has increased over the last few decades to predict target variables in the past or/and future. In the environmental sciences field, they have been implemented across different departments, addressing approaches with varying levels of difficulty degrees: water (Li et al., 2024), soil (Jasoliya et al., 2024), and air (Rahman et al., 2024). The air matrix is an essential component of environmental sciences due to pollution issues arising from population growth and associated activities (Afifa et al., 2024). In this sense, according to the European Environmental Agency, 12% of the EU-28 urban population was exposed to ozone levels above the value threshold set by European Union legislation in 2016, and 98% based on OMS limits (EEA, 2018).

Tropospheric ozone (hereafter O_3_) is a trivalent oxygen molecule (Bagal et al., 2020) and a highly reactive, strongly oxidative gas (Nuvolone et al., 2018), making it a highlighted compound in the mixture of air pollutants. It is a secondary air pollutant formed by photochemical reactions (Galán-Madruga et al., 2024; Xian et al., 2024), mainly from primary air pollutants (Arslan, 2023; Cheng et al., 2023). Well-supported scientific evidence has linked adverse effects in humans to ambient O_3_ exposure (Bromberg, 2016; Chen et al., 2021; Holm & Balmes, 2022; Wang et al., 2025). In this regard, O_3_ exposure is considered a leading contributor to premature mortality, to the current ambient O_3_ concentrations in Europe (Achebak et al., 2024). So, a 9.97 µg/m^3^ increase of O_3_ (expressed to 20 °C and 1013 hPa) is associated with 19.8% increase in mortality (Schwartz et al., 2021).

Building on the previous argument, the European Union developed Air Quality Standards to protect human health and the environment from exposure to air pollutants, designating O_3_ as a criteria pollutant subject to mandatory monitoring in the member states. Recently, a new Air Quality Standard was published in the Official Journal of the European Union dated November 20, 2024 (Directive (EU) 2024/2881). This directive modernizes and reformulates the requirements in previous legislation (Directive 2004/107/EC; Directive 2008 /50/EC) in line with emerging scientific knowledge and the European Union’s proposed environment targets. For O_3_, a target value of 120 µg/m^3^ concentration is set to protect human health, expressed as an average maximum daily 8-h value. This concentration should not be exceeded on more than 18 days per year, averaged over 3 years. In the same frame, a concentration of 100 µg/m^3^, expressed as an average annual level for a maximum daily 8-h period (without being exceeded on more than 3 days per year), is set as a long-term objective to protect human health. In addition, 240 and 180 µg/O_3_ m^3^ (corresponding to an averaging period of 1-h period) are established as alert and information thresholds, respectively (see Annex I of Directive (EU) 2024/2881). O_3_ also has damage effects on vegetation (Ye et al., 2026).

Globally, scientific evidence suggests that tropospheric O_3_ levels have increased over the twentieth century, in line with a global rise in fossil fuel combustion, according to the Intergovernmental Panel on Climate Change (Stocker, 2014). Ambient O_3_ levels were duplicated in Europe between the 1950s and 2000 (Cooper et al., 2014). Ambient air ozone distribution depends on physics (orography, topography, land use planning, among others) (Bhattarai et al., 2024; Wang et al., 2020), meteorological features (emphasizing temperature and solar radiation) (Mousavinezhad et al., 2021), and chemical factors (presence of primary pollutants such as nitrogen oxides, volatile organic compounds, mainly) (Lin et al., 2025; Zhang et al., 2026). This last factor is highly relevant since they field tropospheric O_3_ (Young et al., 2013). Unlike nitrogen oxides and volatile organic compounds, implementing preventive and restrictive measures may do not reduce ambient air O_3_ levels according to numerous scientific studies (Galán-Madruga, 2023; Hashim et al., 2021). Based on that, the difficulty of controlling ambient O_3_ levels, along with the limited O_3_ measurements provided by air quality monitoring networks, makes short-term O_3_ estimation models highly justified. This reflection is relevant, as rising O_3_ levels are a significant public health issue in EU-28 cities (Sicard et al., 2021).

For this reason, this study aims to develop a standard mathematical expression that explains the relationship between urban O_3_ levels and the predictor variables, enabling estimation of daily urban ambient air O_3_ levels. The current work’s contribution is to test the performance of the engineered mathematical formula at both temporal and geographical scales, which differs from previous studies, which did not assess its operation and efficiency using data not included in the proposed approach or its application in other geographical areas. For that, three major European capitals were selected as case studies to achieve that objective, using a vast dataset (2003–2022) comprising both dependent and predictor 24 variables. The suggested approach in this research will reliably enable estimating daily O_3_ levels in non-measured urban zones, decreasing uncertainty on population exposure levels and favoring developing studies conducted by other investigation groups on climate change since O_3_ is regarded as a greenhouse gas (Shah et al., 2024), and short/long-term epidemiologic studies on potential effects of tropospheric ozone on population (Calle-Martínez et al., 2023), among others.

## Existing scientific investigation related to the proposed objective

Within the frame of the research proposed in the current study, notorious research groups have developed mathematical models to estimate urban ambient air O_3_ levels, providing data on ambient O_3_ in areas not monitored, using either statistical or deterministic models (Mghouchi et al., 2024). In the first case, Özbay et al., (2011) employed a multivariate analysis to predict O_3_ concentrations of 1 h later in the ambient air of the Dilovasi region, Turkey. They regarded air pollutants and meteorological variables commonly measured by air quality networks, obtaining adjusted coefficient of determination values of 0.90, 0.85, and 0.92 for annual periods, cooling and warming seasons, respectively. As a limitation, the covered period for setting the original dataset was very short (1 year), and they did not use metrics to assess the performance of the proposed approach. Employing the same approach, de Oliveira et al., (2021) estimated O_3_ levels using air pollutants and meteorological data monitored from 2014 to 2018, individually and combined, obtaining an improved O_3_ estimate when combining both datasets compared with only meteorological variables (15% improvement). Coefficient of determination values of 0.88, 0.91, and 0.63 were observed for three fixed air quality monitoring stations in Munich. They used the root-mean square error of calibration and prediction (expressed in absolute value) to evaluate the model performance, but they were not expressed in percentage value. Other research groups implemented more complex models based on artificial intelligence algorithms (Moncy & Mathew, 2026). They used air pollution and meteorological data from 2020 to 2023 to estimate tropospheric O_3_, but performance metrics were not considered. So, Hosseinpour et al., (2024) combined a multivariate linear regression model with a machine learning algorithm (random forest) to estimate background O_3_ using ground-level observations and satellite data. Note that background O_3_ levels do not correspond to tropospheric O_3_ concentrations. Other investigation groups optimized various machine learning models using Bayesian techniques to accurately forecast ground-level O_3_ concentrations 24 h in advance (Hastie et al. 2009; Troncoso-García et al., 2024). They did not assess the model’s performance. In the same vein, recent comparative studies of machine learning for predicting ground-level O_3_ in Beijing agree that tree-based ensemble models, especially XGBoost, offer the best balance between accuracy and computational efficiency (Liu et al., 2025). They did not assess the model’s performance. A notorious investigation study provided a comprehensive synthesis of recent advancements in the frame of applying machine learning to estimate tropospheric O_3_, highlighted critical challenges, and proposed actionable pathways to further advance in this context (Hickman et al., 2025). Within this context, Li et al., (2023) projected near-surface O_3_ concentrations over Asia for 2020–2100 using a machine learning method with multisource data, although they did not assess the performance of this projection. Ko et al., (2022) predicted O_3_ concentrations using machine learning techniques using measurement and planetary boundary layer data. Based on the results, they concluded that the planetary boundary layer variable improved predictions. Note that in the current study, planetary boundary layer was considered as an input variable in the proposed framework. Another application of deep learning was proposed by Jiménez-Navarro et al., (2024). They provided a novel methodology called time selection layer in deep learning models. Although they reached interesting outcomes, the suggested approach was not validated.

The previously described investigations made valuable advances in the science of ambient O_3_ modeling and introduced interesting methodological approaches. Together with the individual limitation reported in the previous paragraph, to highlight that (i) the most of those studies did not assess the performance of the proposed approaches using well-known metrics, (ii) they did not provide the mathematical formulae resulting the proposed methodological approaches, which makes its application impossible for other investigation groups in other geographical areas, (iii) they did use relatively short periods to develop their approaches, and (iv) they did not validate the employed methodological framework in terms of spatial and geographical, which is essential in modeling approaches to ensure a reliable application in different temporal-spatial scenarios. This feature presents a notable limitation of the previously cited methodologies. In this sense, the present study conducts a deep validation process, evaluating the temporal and spatial impact of the proposed approach. In the case of O_3_, this assessment is paramount, as O_3_ concentrations vary by location worldwide (Denby et al., 2010; Meng et al., 2022).

## Motivation, novelty, and limitations of the suggested approach

Motivation: Legislative efforts to control air pollutant levels and ensure clean air quality have not prevented a rise in tropospheric ozone levels in Europe over time. Developing statistical methods to anticipate exposure to tropospheric ozone is crucial for public health and enables the implementation of prompt corrective measures.

Novelty: Although notable studies have investigated models for estimating urban O_3_ levels, the novelty of the current research lies not in the step-by-step proposed methodological framework, but in assessing and quantifying its performance at both temporal and spatial levels, which is essential for evaluating the efficiency of any estimating model or approach. In this sense, the temporal validation covered periods not included in the development of the generated mathematical formula. On the other hand, spatial validation was conducted in a distinct geographical area from those used to develop the mathematical formula. This fact is significant because estimation models should be useful regardless of time and place, thereby maintaining their validity and reliability.

Limitations: Firstly, there are no limitations on the original dataset’s accessibility, as it can be freely downloaded (open access; please see “Material and Methods”). Nevertheless, it is relevant to highlight that reanalysis datasets (from satellite data) were used instead of independent observational data, which may be a significant limitation in terms of pollution data, as satellite sensors measure a total column of a particular air pollutant, and it translates these concentrations into at the background level may carry uncertainties associated with the proposed approach, as well as a lack of spatial representativeness. Similarly, meteorological data reanalysis does not include phenomena at the microscale level. Secondly, the statistical approach employed in the current study has no limitations due to its simplicity to implement. As a major limitation, the proposed methodological approach yields only one pollution level per city; therefore, this methodology may be applied to provide indicative pollution information in urban environments, providing anticipated knowledge to environmental managers, which is essential for anticipating critical pollution scenarios.

## Material and methods

### Case study and original dataset collection

As a case study for achieving the proposed objective, three major European capitals were selected: Madrid (Spain), Stockholm (Sweden), and Rome (Italy). It is necessary to highlight that the selected cities are placed in highly separated geographical areas (Madrid: 40° 24′ 59.4″ N, 3° 42′ 9.22″ W, Stockholm: 59° 19′ 57.29″ N, 18° 3′ 53.64″ E, 26° 6′ 22.54″ E, and Rome: 41° 53′ 30.95″ N, 12° 30′ 40.79″ E, according to https://latitudelongitude.org/), thereby offering sturdiness to reached mathematical models, which favors implementing deep validation process since diverse ambient O_3_ concentration sceneries are involved. According to World Map of Köppen − Geiger Climate Classification (Kottek et al., 2006), in terms of climate, Madrid is characterized by warm temperate and dry winters and hot summers (code: Csa); Stockholm is marked by snow as a main climate, summer dry precipitation, and warm summer temperature (code: Dfb); and Rome is described by a warm temperate, summer dry and hot summer temperature (code: Csa).

Since meteorological conditions play a fundamental role both in the atmospheric stability processes (Goyal & Jaiswal, 2010) and air pollutants dilution and dispersion (Su et al., 2018), 2003–2022 daily meteorological data were downloaded from ERA5 (ECMWF Reanalysis v5, https://cds.climate.copernicus.eu/datasets/reanalysis-era5-single-levels?tab=overview) re-analysis daily based data. The dataset provided by ERA5 has a spatial resolution of a regular latitude–longitude grid with a spacing of 0.25 degrees (⁓31 km) and a temporal resolution of hourly (https://cds.climate.copernicus.eu/datasets/reanalysis-era5-single-levels?tab=overview). ERA5 reanalysis data is produced by Copernicus Climate Change Service (C3S) at European Centre for Medium-Range Weather Forecasts (ECMWF). In the same context, selected meteorological features were temperature (T), wind direction (WD) and speed (WS), relative humidity (RH), pressure (P), precipitation (Pre), planetary boundary layer height (PBLH), and downward UV radiation at the surface (DRad). On the other hand, a vast dataset (2003–2022) of daily air pollutants was acquired from the Copernicus Atmospheric Monitoring Service (CAMS, https://atmosphere.copernicus.eu/cams-air-quality-data-all-seasons). This dataset is generated by the European Centre for Medium-Range Weather Forecasts (ECMWF) and provides a global reanalysis of atmospheric composition. The dataset provided by CAMS has a spatial resolution of a regular 0.75° × 0.75° latitude–longitude grid (⁓81 km) (https://www.ecmwf.int/en/research/climate-reanalysis/cams-reanalysis?utm_source=chatgpt.com) and the data are available as 3-hourly analyses (Innes et al., 2019). Among the monitored compounds, they were considered air pollutants that participate in O_3_ formation processes. So, O_3_ as a dependent variable, and criteria air pollutants (nitrogen dioxide NO_2_, nitrogen oxide NO, carbon monoxide CO, sulfur dioxide SO_2_, airborne particulate matter (PM_10_, PM_2.5_, and PM_1_, Glenn et al., 2022; Liu et al., 2023), organic compounds (methane CH_4_, ethane C_2_H_6_, acetone C_3_H_6_O, formaldehyde CH_2_O, propane C_3_H_8_, and methanol CH_3_OH, Chutia et al., 2019; Pfister et al., 2019; Sinha et al., 2014), and oxidant compounds (peroxyacetyl nitrate C_2_H_3_NO_5_, nitrate ion NO_3_^−^, and methyl peroxy radicals CH_3_O_2,_ Stockwell et al., 1995; Jakobi & Fabian, 1997; Zafar et al., 2018) as predictor inputs. Based on the previous listing, 24 inputs were selected to estimate daily urban ambient air O_3_ concentrations. Understanding the relationships between air pollutants and meteorological variables is essential for controlling and mitigating atmospheric pollution (Kim et al., 2017). The data downloaded from the original sources are open access; therefore, any reader can use these sources to download the data.

### Influence of the selected meteorological features on daily ozone levels

Since meteorological conditions play a pivotal role in atmospheric pollution stability (Liu et al., 2020), evaluating their impact on local O_3_ levels is essential in air quality studies. The objective will be to elucidate the influence of weather variables on O_3_ pollution. Daily meteorological data were gathered to address a global (across the three cities) and individual (for each city) approach, ultimately executing a normalization process for each target meteorological variable to establish a comparable scale. Then, the SMOreg regressor (Sequential Minimal Optimization Regression), a machine learning algorithm, was applied to evaluate the previously cited objective. This algorithm employs radial basis function kernels to train a support vector regression (Mohammed & Fernandez, 2023), with the selected Polykernel in line with other studies (Xu, 2022). O_3_ and meteorological conditions are the dependent and independent variables, respectively.

### Influence of the covered air pollutants on daily ozone levels

Since O_3_ formation depends on other air pollutants, the influence of each pollutant on O_3_ levels was evaluated. To establish the influence of each selected air pollutant on tropospheric O_3_, a correlation analysis (COA) was conducted to understand the individual impact of each compound on the dependent variable. COA measures the two-sided relationship between two variables (Mikheev & Kazakov, 2017). Therefore, it properly reveals the convoluted association between distinct datasets (Zhang et al., 2017). Thus, this associating technique helps understand the connectivity between diverse study variables. In this study, Pearson’s coefficient was used as an evaluation indicator to appreciate the reached outcomes. Strong and weak associations (close to 1 and 0, respectively) are supported by high and low COA values across variables within the same dataset. The interpretation reported by Dancey and Reidy, (2007) was used as a criterion to assign a connectivity level to variables. They organized the potential outcomes of the coefficient of correlation (*r* = 0 to 1) into several categories, assigning each category a relationship value. So, *r* = 0 carries zero relation, if 0.1 < *r* < 0.3 implies a weak relation, if 0.4 < *r* < 0.6 suggests a moderate relation, if 0.7 < *r* < 0.9 indicates a strong relation, and finally, *r* = 1 corresponds to a perfect relation. This technique was applied to each independent variable individually, considering daily observations.

### Combined influence of all covered predictor variables on daily ozone levels

Since ambient air O_3_ levels are a function of the interaction between pollution and weather conditions, a study involving all input variables was conducted to determine their combined influence on the target pollutant. For that, a combined PCA-MLR analysis (principal component analysis-multiple linear regression) was performed to quantify the contribution of each predictor to the dependent variable, while simultaneously accounting for the predictor dataset. PCA is a classic chemometric technique that applies a rotational algorithm to rank an original dataset comprising possibly correlated variables into a smaller set of linearly uncorrelated variables (Galán-Madruga, 2021). The new sub-dataset of variables captures most of the information from the original dataset (Li et al., 2019). In this study, the varimax method and eigenvalues > 0.8 (the Kaiser Criterion) were used to select principal components (PCs) that accounted for most of the cumulative variance. In addition, a cumulative variance exceeding 80% was observed for the subsequent PCs. Subsequently, the outcomes from the PCA were used to perform an MLR analysis to quantify the contribution of each predictor variable to the PCs. A particular predictor input with a high contribution indicates it is relevant to the dependent variable. All predictor variables were considered to develop the proposed model.

### Modeling urban ambient air daily ozone levels from selected predictor inputs

The original meteorological and air pollutant dataset (2003–2022) was divided into two sub-clusters to engineer the target mathematical model. The first cluster covered 2004–2021 and was used to develop the modeling approach for this study, while the second cluster encompassed 2003 and 2022 to validate the generated approach.

In this study, a multivariate analysis is applied using a comprehensive multiple linear regression analysis to incorporate all variables (dependent and 24 independent inputs). The resulting expression will establish the relationship between the dependent and independent variables, elucidated through a mathematical model (Kumar et al., 2017), and estimate urban ambient air daily ozone concentrations from the selected predictor variables. The mathematical formulae explaining that relationship is reported by Eq. (1):

1 $${\mathrm{O}}_{3}=Cte+\sum_{i=1}^{n}{a}_{i}{x}_{i}$$

 

where ${\mathrm{O}}_{3}$ is urban daily ozone concentration, $Cte$ corresponds to a constant value (intercept), $a$ represents the regression coefficients for each predictor input, $x$ equals the slope of each selected predictor input from the original dataset, and $n$ corresponds to the number of selected predictor inputs ($n$ =24). No identifying outlier methods were applied to the original dataset (2004–2021, *N* = 19,725).

Since O_3_ formation involves complex photochemistry, its production is not proportional to emissions of primary air pollutants, although it is important to note that this non-linear relationship between O_3_ and its precursors can vary across geographical areas, given that the presence of precursor pollutants in the ambient air depends on topography, emission sources, and meteorological conditions. Despite that, multivariate linear analysis is fully justified when the goal is to model complex relationships among multiple variables, even in the presence of potential collinearity (Kim, 2019), without invalidating the analysis (Mason et al., 1991). Yielding O_3_ carries nonlinear dynamics between O_3_ and VOCs/NOx, which have already been explained in the scientific literature (Chameides et al., 1992; Seinfeld & Pandis, 2016). Investigation studies demonstrate that machine learning models exhibit greater predictive accuracy than linear regression in estimating O_3_ concentrations (Jumin et al., 2020; Zhou et al., 2023). Given that the application of machine learning techniques in air quality may carry methodological problems such as generalization (Hickman et al.., 2025), variable selection, and inadequate validation strategies (Tang et al., 2024), and it can also generate technical problems such as overfitting, lack of interpretability, and parameterization difficulties (Lu & Wang, 2014), the current study aims to provide an easy-to implement methodological framework to estimate daily urban O_3_ concentrations using multiple linear regression analysis. Linear models provide clear interpretations and allow us to understand how dependent variables change in response to changes in the predictor variables. In addition, they are easiest to communicate and reproduce models (James et al., 2023). The scientific community has already accepted the utilization of multivariate linear analysis techniques to estimate O_3_ concentrations (Abdul-Waha et al., 2005; Iglesias-González et al., 2020; Sousa et al., 2006), which justifies its employment in the current study.

In this context, the World Meteorological Organization urges to implement transparent and reproducible models in operational studies (WMO, 2008).

Based on the previous explanation, it is fundamental to highlight that the use of a simple methodology does not entail rejection, but rather the opposite, given that the objective of science is to improve the quality of life of the population and facilitate their daily tasks. Applying complex models does not necessarily yield better results than applying simple models.

### Validating the proposed modeling approach

Applying a general estimate model must be suitable at any time. To test this hypothesis, the validity of the suggested model was assessed in 2003 and 2022 (note that these years were not employed in model development). For that, urban daily O_3_ concentrations were estimated for the covered cities (1 January and 31 December 2003 and 2022) using the standard mathematical expression derived from the modeling process.

The confidence of the proposed estimate modeling was evaluated by performing a simple linear regression analysis of 2003 and 2022 estimated vs. current daily O_3_ concentrations. The following assessment requirements were regarded: (i) the correlation measurement between estimated vs. actual daily O_3_ concentrations, (ii) statistical significance of the simple linear regression expression with one-way ANOVA, (iii) statistical significance of the regression coefficient of the independent variable, and (iv) statistical significance between estimated vs. actual concentrations using a *t* test (Yang et al., 2018).

In addition, other statistical indicators frequently used in atmospheric sciences (Karunasingha, 2022) were considered to evaluate the recommended estimated model performance, namely, root mean square error (RMSE), mean prediction error (MAE), and mean absolute percentage error (MAPE). They were estimated based on other previous scientific studies (Galán-Madruga et al., 2025).

Once the temporal validity of the suggested mathematical model for estimating daily O_3_ concentrations in urban ambient air was tested, a geographical analysis was conducted to determine whether the recommended approach can be applied effectively across urban locations. For that, a new urban environment was selected. In this case, Bucharest city was used to test the validity of this last hypothesis. In this sense, urban daily O_3_ concentrations from 2004 to 2021 (18 years) were estimated using an equation relating to the dependent variable (O_3_) to 24 independent inputs selected for the estimation. Then, these estimated daily O_3_ concentrations were compared with the current daily O_3_ concentrations.

### Statistical treatment

Normality and variance homogeneity were assessed using the Kolmogorov–Smirnov and Levene tests, respectively. ANOVA or Kruskal–Wallis tests were conducted on normally or non-normally distributed data, respectively. *p* < 0.05 values were considered statistically significant.

## Results

### Air quality status in the target European cities

**Fig. 1:**
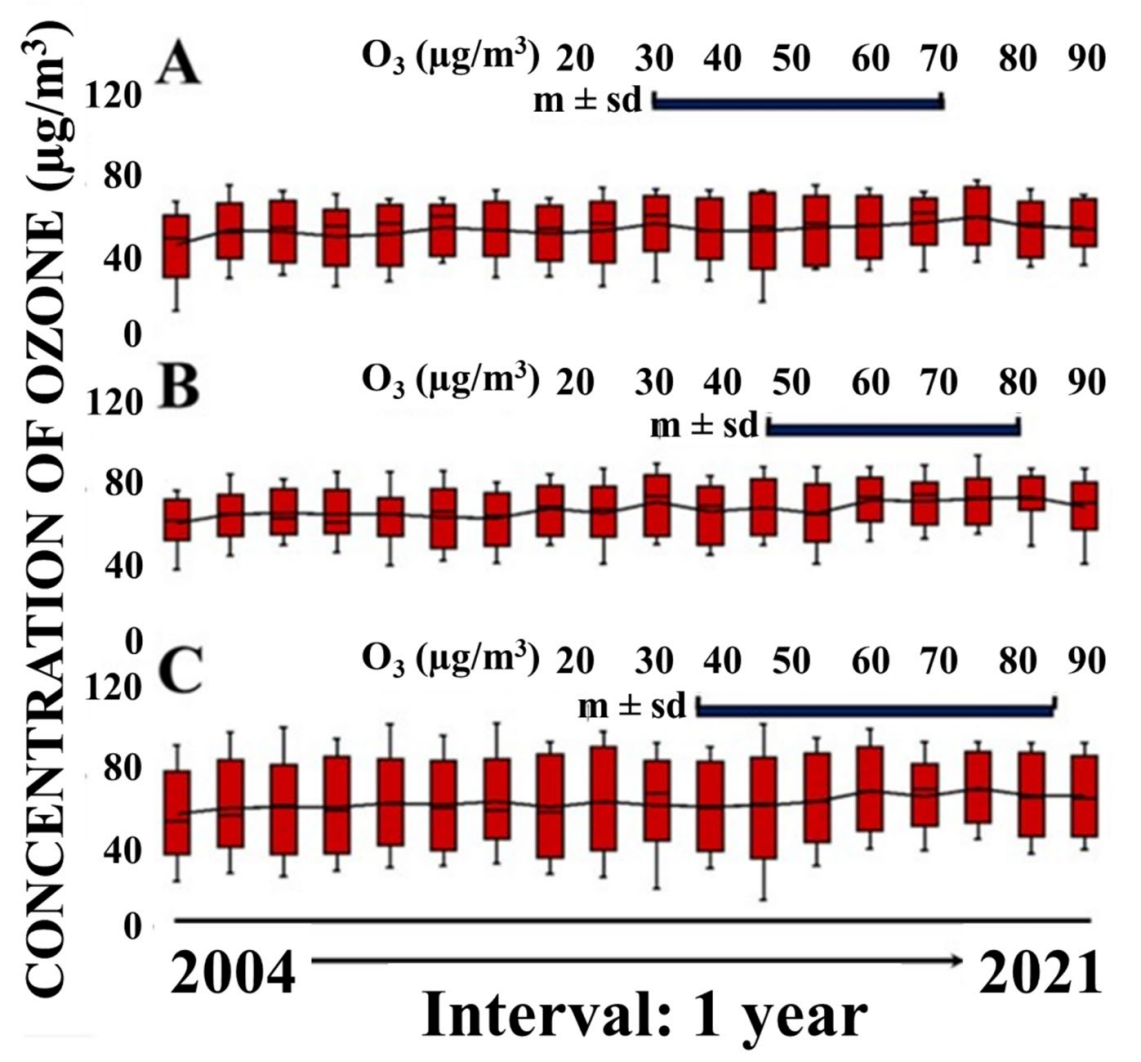
Studying 2004–2021 ozone concentration trends in the researched European cities. **A** corresponds to Madrid, **B** stands for Stockholm, and **C** is Rome

To provide an overview of the polluting scenery over the investigated period in each selected city, an air quality study focused on ozone levels was conducted. For that, daily pollution data was used according to “Material and Methods”. This information is vital to demonstrate that the proposed methodological approach covers different pollution scenarios. Figure 1 shows the trend in the O_3_ levels in the researched cities from 2004 to 2021. For each evaluated city, annual O_3_ concentrations are reasonably similar over the studied period, and each city shows the same profile. Nevertheless, quantitative differences are exhibited. So, 2004–2021 average concentrations of 52.32 µg O_3_/m^3^ (95% CI 51.88–52.77 µg O_3_/m^3^, standard deviation 18.98 µg O_3_/m^3^, range 6.14–107.78 µg O_3_/m^3^), 65.19 µg O_3_/m^3^ (95% CI 64.78–65.80 µg O_3_/m^3^, standard deviation 17.04 µg O_3_/m^3^, range 4.53–117.98 µg O_3_/m^3^), and 62.00 µg O_3_/m^3^ (95% CI 61.41–62.60 µg O_3_/m^3^, standard deviation 24.54 µg O_3_/m^3^, range 5.60–129.12 µg O_3_/m^3^), were observed in Madrid, Stockholm, and Rome, respectively.

### Influence of the selected meteorological features on daily ozone levels

Since meteorological conditions vary across the studied cities, studying their influence is justified. In this sense, the 2004–2021 numeric expression, considering the three targeted cities, linking O_3_ and meteorological variables points to Pre as the most dominant weather variable, followed by WS, and DRad (positive influences), respectively (O_3_ = 0.1233 × T + 0.0917 × WD + 0.3368 × WS − 0.0703 × RH + 0.2093 × P + 0.4951 × Pre + 0.3102 × PBLH + 0.3267 × DRad − 0.1381, *r* = 0.798, number of kernel evaluations: 1,373,909,304, and total number of instances: 26,300). A individual study exhibited PBLH as the most dominant weather variable followed by Pre, and WS (positive influences), respectively, in Madrid (O_3_ = 0.0118 × T − 0.0033 × WD + 0.2376 × WS + 0.0699 × RH − 0.1111 × P + 0.3749 × Pre + 0.4920 × PBLH + 0.2364 × DRad + 0.1312, *r* = 0.886), DRad as the predominant variable followed by WS and Pre (positive influences) in Stockholm (O_3_ = 0.0123 × T + 0.0110 × WD + 0.2101 × WS + 0.0640 × RH − 0.0148 × P + 0.1891 × Pre + 0.1043 × PBLH + 0.3660 × DRad + 0.4991, *r* = .7832), and PBLH as the prevalent feature followed by Pre and DRav (positive influences) in Rome (O_3_ = 0.3165 T + 0.0430 × WD + 0.1082 × WS − 0.2290 × RH − 0.2165 × P + 0.3484 × Pre − 0.3908 × PBLH + 0.3427 × DRad + 0.078, *r* = 0.9211).

### Influence of the covered air pollutants on daily ozone levels

Table 1 shows the individual relationships between daily O_3_ levels and each air pollutant, expressed as Pearson’s correlation coefficients, for each city (Madrid, Stockholm, and Rome). Since tropospheric O_3_ is a secondary pollutant (it is not directly emitted into the atmosphere but formed by photochemical reactions among other primary pollutants, Derwent, 2020), the NOx/VOCs ratio determines atmospheric O_3_ chemistry (Nguyen et al., 2022). NOx includes NO and NO_2_, although NO reacts within minutes with O_3_ to yield additional NO_2_ (Lin et al., 2016). Regarding VOCs, their role in tropospheric O_3_ formation points toward the NO oxidation to NO_2_ (Finlayson-Pitts & Pitts, 1993). Based on this argument, NOx and COV are identified in all cities as dominant features exhibiting a correlation higher than 0.5 (mainly NO_2_). In addition, other compounds, such as CO and peroxy radicals, appear on this checklist. In the case of CO, it aids in producing ozone, influencing global ozone concentrations by affecting OH and HO_2_ concentrations (Latha & Badarinath, 2004), and, according to Wedow et al., 2021, peroxy radicals are also directly responsible for tropospheric O_3_ formation. Broadly, the individual assessment of predictor inputs is moderated by atmospheric O_3_ chemistry.

**Table 1:** Pearson’s coefficient of correlation value obtained when applying correlation analysis between the dependent variable (O_3_) and each air pollutant listed as a predictor input

	Madrid			Stockholm			Rome		
Predictor input	*r*^a^	Lower CI^b^	Upper CI	*r*	Lower CI	Upper CI	*r*	Lower CI	Upper CI
NO_2_	**0.814**	0.798	0.894	**1.000**	**–-**	**–-**	**1.000**	**–-**	**–-**
NO	** − 0.579**	** − **0.587	** − **0.570	** − **0.272	** − **0.294	** − **0.250	** − 0.583**	** − **0.599	** − **0.567
Nitratos	** − **0.063	** − **0.075	** − **0.051	** − **0.070	** − **0.094	** − **0.046	** − **0.092	** − **0.116	** − **0.068
Peroxyacetyl nitrate	**0.552**	0.544	0.561	0.369	0.348	0.390	**0.760**	0.750	0.770
CO	** − 0.638**	** − **0.645	** − **0.630	** − 0.569**	** − **0.585	** − **0.552	** − 0.710**	** − **0.721	** − **0.697
SO_2_	** − **0.455	** − **0.465	** − **0.446	** − **0.135	** − **0.158	** − **0.111	** − **0.181	** − **0.205	** − **0.158
PM_10_	** − **0.405	** − **0.415	** − **0.395	0.043	0.019	0.067	** − **0.410	** − **0.430	** − **0.390
PM_2.5_	** − **0.408	** − **0.418	** − **0.398	0.028	0.004	0.052	** − **0.411	** − **0.430	** − **0.390
PM_1_	0.063	0.051	0.075	0.109	0.085	0.133	0.057	0.033	0.081
Methane	** − **0.286	** − **0.297	** − **0.275	** − **0.465	** − **0.483	** − **0.445	** − 0.756**	** − **0.766	** − **0.745
Acetone	0.058	0.046	0.070	0.113	0.089	0.137	0.037	0.012	0.061
Ethane	** − 0.503**	** − **0.512	** − **0.494	** − **0.373	** − **0.394	** − **0.352	** − 0.642**	** − **0.656	** − **0.627
Formaldehyde	0.342	0.331	0.352	0.441	0.421	0.460	**0.668**	0.654	0.681
Propane	** − 0.555**	** − **0.563	** − **0.546	** − 0.600**	** − **0.615	** − **0.584	** − 0.689**	** − **0.701	** − **0.676
Methanol	0.416	0.406	0.426	0.394	0.374	0.414	**0.629**	0.614	0.643
Peroxy radicals	**0.662**	0.655	0.669	**0.565**	0.548	0.581	**0.786**	0.776	0.795

^a^Pearson’s coefficient of correlation

^b^Confidence interval

Values  in bold highlight the most significant correlation coefficients (r0.500)

### Combined influence of the covered predictor variables on daily ozone levels

Given that the target air pollutants and meteorological features do not individually affect tropospheric O_3_ formation, a combined influence study encompassing all predictor inputs is justified. The combined action of the independent variables may modify the individual impact of each predictor on the dependent variable. In this sense, Tables S1–S3 reveal the outcomes obtained when implementing the PCA technique in each selected city. As shown, a minimum of 7 and maximum of 9 PCs were defined (8 PCs for Madrid city, 9 for Stockholm city, and 7 for Rome city), with cumulative variances of 83.38, 82.62, and 83.00% for Madrid, Stockholm, and Rome, respectively. Note that PC1 corresponds to the most dominant principal component, as it explains most of the original information in the primary dataset, accounting for 33.05, 28.96, and 37.50% of the total variance for Madrid, Stockholm, and Rome, respectively (see Table S4), with reasonably similar profiles across all cases.

The results obtained from the PCA technique were used to conduct an MLR analysis. To quantify the total weight of each predictor, the partial contributions for each predictor and each PC were summed (see the outcomes in Tables from S5 to S7). Then, the mean–variance value was calculated each independent variable’s total variance set to identify the most representative predictor inputs. In this context, the individual predictor variables with total variances above the average value exhibit the highest representativeness. So, mean–variance values of 3.47, 3.44, and 3.46% were obtained in Madrid, Stockholm, and Rome, respectively.

### Modeling urban ambient air daily ozone levels from 24 predictor inputs

**Fig. 2:**
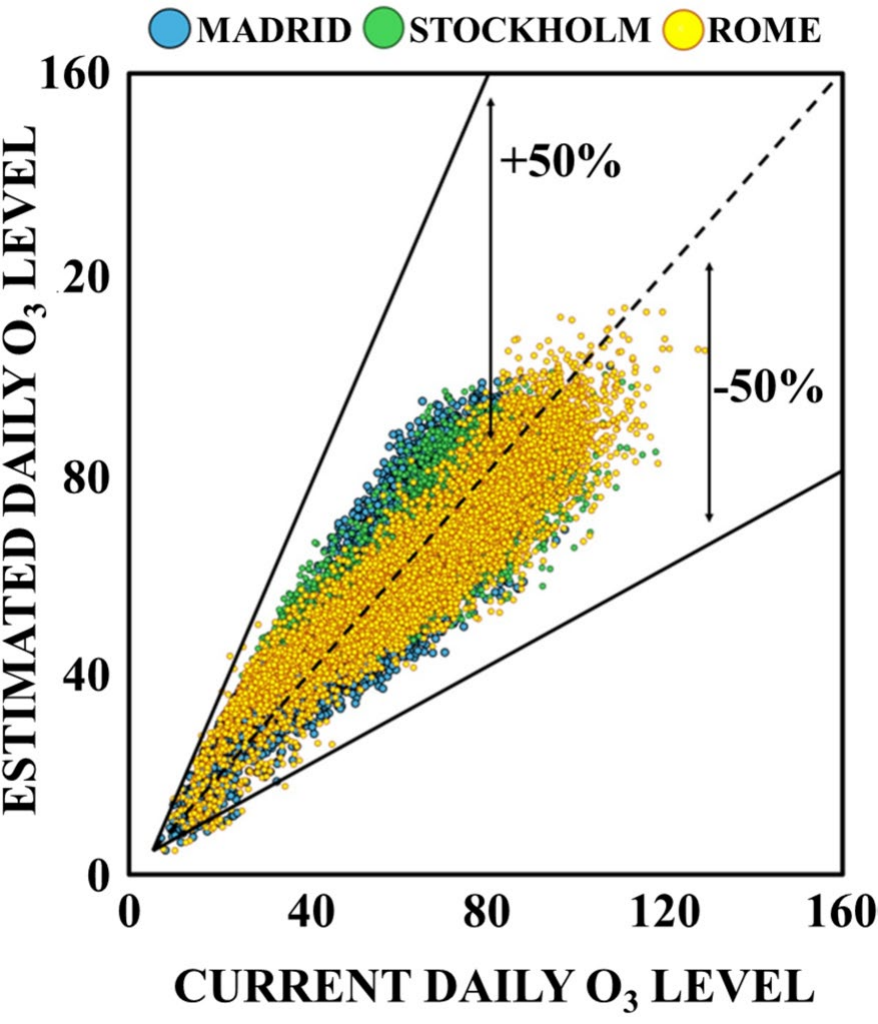
Scatterplot of 2004–2021 estimated vs. current daily O_3_ concentrations (*n* = 26,300 data for the dependent variable and for each 24-predictor input). Units: µg/m.^3^

Although PCA revealed potential collinearity among the independent variables with respect to O_3_, all input variables were considered in this study, as Özbay et al. (2011) observed worse performance when removing input variables with linear associations. The multivariate analysis described in “Material and Methods” yielded a standard mathematical expression for estimating urban daily O_3_ concentrations from predictor variables. Table 2 shows the coefficients of the multiple linear regression equation for each predictor, as specified in Eq. (1) during the estimation process. All data included in the 2004–2021 original dataset were used to develop the proposed methodology. According to the standardized beta coefficients, the predictor variables with positive coefficients have a direct association with the dependent variable (NO, peroxyacetyl nitrate, PM_10_, ethane, methyl peroxy radicals, WD, WS, P, Pre, PBLH, and Drad), and those variables with negative coefficients present an inverse relationship (rest of input variables). Regardless of the type of association with the dependent variable, particulate matter (PM_10_ and PM_2.5_ with values of 1.873 and − 1.837, respectively) exhibits the greatest relative influence of all predictor variables. Figure 2 pictures a scatterplot of estimated vs. current daily O_3_ concentrations over the study period. Note that the generated mathematical formula maintains a good predictive capacity (Pearson’s coefficient of correlation equals 0.922, goodness of fit of 84.99%). Applying a general multiple regression analysis yielded a linear equation with a slope closer to 1 (0.86) and an intercept closer to 0 (6.81). It is relevant to highlight that the range of daily O_3_ concentrations spans from 2 to 120 µg/m^3^, in order to emphasize the validity of the presented regression model.

**Table 2:** Coefficient of the multiple linear regression equation for each predictor variable used to estimate urban daily O_3_ levels

Predictor variable (unit)	Regression coefficients	Standardized beta coefficients	Error deviation	*p* value
Cte	** − **79.959		13.01	< 0.001
NO_2_ (µg/m^3^)	** − **0.001	** − **0.009	0.000	0.003
NO (µg/m^3^)	0.088	0.030	0.015	< 0.001
NO_3_^−^ (µg/m^3^)	** − **0.193	** − **0.003	0.140	0.168
Peroxyacetyl nitrate (ppb)	7.179	0.303	0.112	0.000
CO (ppb)	** − **0.075	** − **0.360	0.002	0.000
SO_2_ (µg/m^3^)	** − **0.599	** − **0.156	0.019	< 0.001
PM_10_ (µg/m^3^)	2.170	1.873	0.058	< 0.001
PM_2.5_ (µg/m^3^)	** − **2.953	** − **1.837	0.081	< 0.001
PM_1_ (µg/m^3^)	** − **0.001	** − **0.035	0.000	< 0.001
Methane (ppb)	** − **0.005	** − **0.016	0.008	0.541
Acetone (ppb)	** − **0.001	** − **0.026	0.000	< 0.001
Ethane (ppb)	13.008	0.227	0.380	< 0.001
Formaldehyde (µg/m^3^)	** − **0.106	** − **0.007	0.171	0.534
Propane (ppb)	** − **6.141	** − **0.243	0.150	0.000
Methanol (ppb)	** − **0.334	** − **0.100	0.026	< 0.001
Methyl peroxy radicals (ppb)	1124.052	0.149	52.940	< 0.001
T (K)	** − **0.064	** − **0.026	0.046	0.164
WD (degrees)	0.014	0.065	0.001	< 0.001
WS (m/s)	1.809	0.125	0.058	< 0.001
RH (%)	** − **3.59 10^**−**14^	** − **0.001	0.000	0.700
P (millibars)	0.142	0.190	0.011	< 0.001
Pre (mm/day)	0.394	0.060	0.018	< 0.001
PBLH (m)	0.008	0.102	0.000	< 0.001
Drad (J/m^2^)	2.220	0.381	0.031	0.000

To elucidate whether the suggested estimate modeling is adequate in terms of legislation, the data quality objectives set by the current European legislation for modeling applications were considered, as applicable under the 2021 Directive 2008/50/EC. This European air quality standard sets data quality objectives for ambient air quality assessment concerning modeling uncertainty, among other aspects. In the case of O_3_, it establishes short-term modeling uncertainties of 50% for both hourly and 8-h averages (see Annex I of Directive 2008/50/EC). Since legislation does not specify a daily O_3_ modeling uncertainty, the short-term value is used in this research. A practical assessment of the relationship between a candidate vs. reference method leads to the evaluation of accuracy as a metrological reference value. It is the agreement closeness between a measured and true value (Fuentes-Arderiu & Rigo-Bonnin, 2012), and it was calculated based on Eq. (2) (Eurachem, 2012).

2 $$Ac=\frac{\left({O}_{3C}-{O}_{3E}\right)}{{O}_{3C}}$$

where $Ac$ is the accuracy expressed in %, ${\mathrm{O}}_{3C}$ equals the current O_3_ concentration, and ${\mathrm{O}}_{3\mathrm{E}}$ equals the estimated O_3_ concentration. Within this context, a 2004–2021 average accuracy of 0.01 µg O_3_/m^3^ was reached (expressed as a relative value, 0.86%). Broadly, long-term average values may not reflect model’s performance, as they may be smoothed. For this reason, the range of daily accuracy values is a proper indicator for testing compliance with legislative requirements. So, the minimum and maximum daily accuracies were − 1.60 µg O_3_/m^3^ (equivalent to 52.34%) and 0.64 µg O_3_/m^3^ (equivalent to 62.47%), respectively. To offer a further perspective, the total number of exceedances of legislative requirements (50% modeling uncertainty) is 21 (0.079%), of which 18 (0.068%) fell within the 50–60% interval and 3 (0.011%) in the interval above 60%. This finding highlights the validity of the proposed approach.

Broadly, given the European Union’s growing concern about atmospheric pollution and the fact that the Air Quality Directives are based on scientific evidence, diverse notable research groups have focused on developing estimation models and researching the underlying O_3_ mechanisms from an empirical perspective (De Marco et al., 2022). In this context, Özbay et al., (2011) applied multivariate statistical methods in annual and seasonal predicting O_3_ using polluting and meteorological data (PM_10_, SO_2_, NO, NO_2_, CO, O_3_, CH_4_, NMHC, temperature, rainfall, humidity, pressure, wind direction, wind speed, and solar radiation) monitored over 1 year. Applying multiple linear regression generated adjusted *r*^2^ values of 0.90, 0.85, and 0.92 for the annual period, cooling season, and warm season, respectively. Note that results were reasonably similar to the current study, even though the estimated period was daily, not yearly or seasonal. Alonso et al., (2025) assessed the atmospheric patterns associated with high O_3_ levels and identified the main ozone precursors on mainland Portugal. For that, they applied a stepwise regression analysis using the following predictors: fire radiative power, thermal amplitude, maximum temperature, boundary layer height, NO_2_ concentration, radiation, and time of the year. *R* results like the current study were found (ranging between 0.92 and 0.93).

The scientific literature reports a wide variety of models for estimating ozone. The first approaches are based on statistical tools, such as logistic regression (Ooka et al., 2011), temporal series streams treatment (Rao & Zurbenko, 1994), and multivariate analysis techniques (Melkonyan & Kuttler, 2012). Most complex models focused on estimating ambient ozone levels, such as deterministic models (Arregocés et al., 2023), based on different physical and chemical mechanisms associated with the emission, transport, and dispersion of atmospheric pollutants (Sharma et al., 2016). In general, deterministic models have low accuracy in micro-urban environments (H. Wang et al., 2022). In addition, these models incur high computational costs and rely heavily on scarce data on emissions and air pollutant sources (Tella et al., 2021). Faced with these inconveniences, it is important to highlight the simplicity of the approach employed in the current study compared to most complex models proposed for predicting O_3_ concentrations.

The notorious previous O_3_ modeling studies have contributed to understanding atmospheric O_3_ chemistry, providing significant advances in the scientific field. Nevertheless, the reached models were not tested in distinct periods compared to those used to develop the models. As an innovative feature, this investigation tests the generated estimating O_3_ model derived from the O_3_/24 predictors relationship across distinct periods, compared with other models.

### Validating the proposed modeling approach

At the scientific level, a fundamental feature of the development of estimation models is the validation process, which assesses the model’s reliability. The more robust the validation process, the higher the reliability of the proposed estimation approach. When evaluating models for ambient air, their validity should be tested for both temporal and geographic applicability, as the target model must be applicable independently of time and location. This last factor is transcendental since topography, emission sources of air pollutants, and weather variables are specific to each geographical area, which justifies the need for geographically testing ambient air pollution estimation models.

**Fig. 3:**
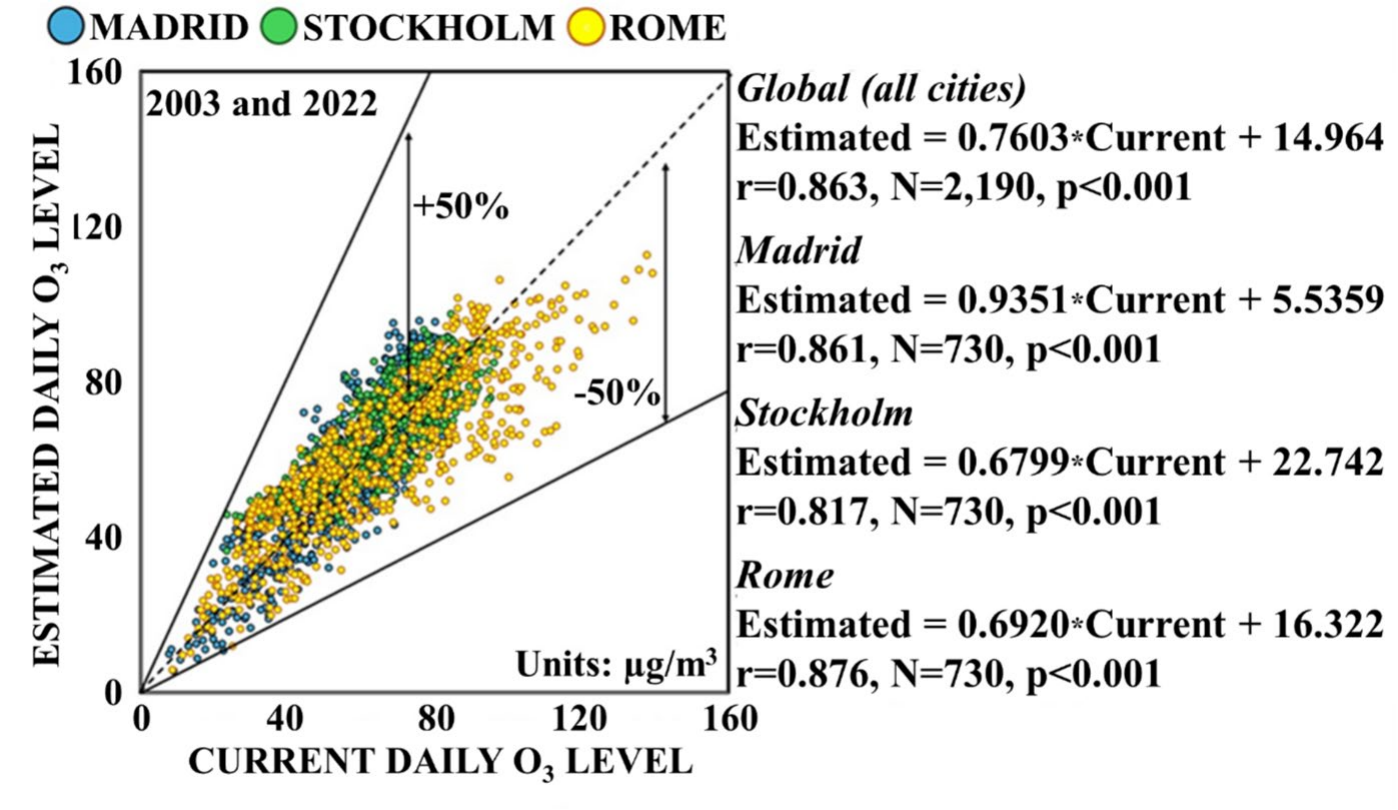
Scatterplot of 2003 and 2022 estimated vs. current daily O_3_ concentrations to test applying the proposed approach regardless of time (*n* = 2190 data for the dependent variable and each 24-predictor input)

In this sense, the proposed predictive model was validated by comparing the daily O_3_ levels estimated from the standard mathematical expression generated by multivariate analysis with the current satellite daily O_3_ measurements, according to Sect. 3.1. To assess the temporal reliability of the proposed estimate model, a linear regression test was conducted (with estimated and actual O_3_ levels as the dependent and independent variables, respectively). The tested period covered 2003 and 2022 in Madrid, Stockholm, and Rome. Note that these years were not employed to develop the proposed model. The global linear regression model reached 0.863 Pearson’s coefficient (O_3_ estimated = 0.76 × O_3_ current + 14.96, *N* = 2190 data for each independent input, *p* < 0.001), thereby sustaining 24 predictor variable set explain 74% of the total dependent variable variance (see Fig. 3). Note that all estimated data falls into the legislative ± 50% interval. Another fundamental assumption in the validation processes using linear regression analysis is that residuals are independence. In this sense, the Durbin–Watson statistic was between 1.5 and 2.5 (1.8), indicating that the residuals were independent.

The ANOVA test of the linear equation generated by linear regression analysis between estimated and current daily O_3_ levels indicated that the prediction model significantly improves forecasting of the dependent variable (O_3_) with an *F* value of 12,295.30 (*p* = 0.00). In addition, ANOVA of the regression coefficient of the independent variables demonstrated their significance and inclusion in the linear regression equation (*p* = 0.00). Non-significant differences (*p* = 0.298) were found when conducting paired samples using the paired *t* test (estimated and current O_3_ concentration). The RMSE, MAE, and MAPE statistical indicators resulted in 1.05 μg/m^3^, 1.11 μg/m^3^, and 11.16%, respectively. It is relevant to note that the range of O_3_ concentrations spans 7 to 139 µg/m^3^. The model’s performance indicators showed RMSE and MAE values of 1.05 µg/m^3^ and 1.11 µg/m^3^, respectively, suggesting a low magnitude of error and a relatively homogeneous residual distribution. The closeness between these two metrics indicates that there is no dominant influence of extreme errors, given that RMSE is more sensitive to large deviations due to the quadratic penalty, while MAE reflects the average absolute error more robustly against outliers (Chai & Draxler, 2014; Willmott & Matsuura, 2005). In relative terms, the MAPE obtained indicates a moderate percentage error level. Nevertheless, this metric should be interpreted with caution, as it tends to be unstable when the observed values are low, leading to an overestimation of the relative error in such cases (Armstrong & Collopy, 1992). This behavior is particularly relevant in this study, given that the O₃ concentrations include minimum values close to 7 µg/m^3^. The observed concentration range (7–139 µg/m^3^) provides context for the model’s absolute error. In this sense, an RMSE close to 1 µg/m^3^ represents a small fraction of the total range, indicating good overall model performance in terms of absolute fit. This type of interpretation based on physical units is consistent with standard practices in environmental model evaluation, where metrics such as RMSE and MAE are widely used due to their straightforward interpretability (Bennett et al., 2013). The combined use of multiple metrics is methodologically recommended, as no single measure fully captures the performance of a predictive model. Different metrics provide complementary information about the error distribution, its scale, and sensitivity to extreme values (Hyndman & Koehler, 2006). In this context, combining RMSE, MAE, and MAPE provides a more robust assessment of model behavior under different concentration conditions.

Despite the proposed approach’s performance being notably high, it is necessary to note that the reanalysis data sustained its engineering, which may have led to an inflated performance, as these data are associated with uncertainties derived from reanalysis models.

## Discussion

In response to the public health risks associated with tropospheric ozone exposure, this study seeks to model daily ozone concentrations in urban environments using air pollution and meteorological data from Madrid, Stockholm, and Rome over the 2004–2021 period. The obtained outcomes, in terms of air quality status, show quantitative differences in O_3_ levels. The geographical location, as determined by latitude and longitude, influences tropospheric O_3_ levels (Elshorbany et al., 2024; Gorai et al., 2017), favoring higher latitudes and downward O_3_ transport from the stratosphere to the troposphere (Fadnavis et al., 2010; Škerlak et al., 2014), which may explain the highest average O_3_ levels in Stockholm. Notable scientific studies concluded that ozone formation, which depends on large-scale radiation and transport, may be more evident in mid- to high latitudes because transported air masses incorporate long-distance precursors (Solberg et al., 2005). In this frame, Fleming et al., (2018) reported that O_3_ monitoring locations in the Southern Hemisphere have lower daily maximum 8-h ozone concentrations than those in the Northern Hemisphere at similar latitudes. In this sense, significant differences are exhibited among the covered cities (*p* < 0.05, Kruskal–Wallis test). This quantitative inequality favors obtaining a standard expression for estimating urban daily O_3_ levels since the air quality status in the researched cities spans different O_3_ polluting scenarios.

Despite the dissimilarity in terms of concentration, a seasonal pattern is conserved, exhibiting the highest O_3_ levels in the warm period (see Fig. S1), with reasonably similar 2004–2021 warm vs. cold period differences for Madrid and Rome (23.24 and 26.82%, respectively), while Stockholm showed a low variation (16.36%). The highest contributions fell into Summer for all cities (Fig. S2), with 2004–2021 average contributions of 32.71, 29.40, and 36.35%, for Madrid, Stockholm, and Rome, whereas those lowest ones corresponded to Winter, with 2004–2021 average contributions of 16.90, 18.90, and 14.58%, for Madrid, Stockholm, and Rome, respectively. It is well known that O_3_ formation is favored by high temperatures (Lu et al., 2021; Pusede et al., 2015). In the case of this study, the highest and lowest temperatures were Summer (298.03, 289.55, and 297.90 K for Madrid, Stockholm, and Rome, respectively) and Winter (279.32, 272.18, and 281.03 K for Madrid, Stockholm, and Rome, respectively), coinciding with the highest and lowest O_3_ concentrations. Within the same context, the hottest months shelter the highest O_3_ concentrations (see Fig. S3).

In terms of climate, the individual influence of meteorological variables on O_3_ levels differs across cities in the combined study. Since the weather features act in combination with O_3_, studying them together revealed a remarkable influence of these variables on local O_3_ concentrations, which aligns with other studies reporting that atmospheric stability may outweight the impact of local pollution sources (Yuval et al., 2020). Note that Prec, WS, and DRad are influential weather variables for local O_3_ levels. The first two variables play a fundamental role in the dilution and dispersion of air pollutants (Mayer, 1999). In contrast, DRad preserves a key function in forming O_3_ processes (Nelson et al., 2023), thereby favoring increased photochemical reactivity (Deng et al., 2019).

Among all predictor variables, they were the most representative for Madrid, Stockholm, and Rome: NO_3_^−^, peroxyacetyl nitrate, CO, methane, formaldehyde, propane, methanol, methyl peroxy radicals, temperature, wind speed, and downward UV radiation at the surface. Nevertheless, those without representation were NO, SO_2_, PM_1_, acetone, pressure, and precipitation.

The proposed approach to estimate daily urban O_3_ levels generates reliability consequently with the performance outcomes reached in the study. To contextualize the results obtained in terms of performance indicators, a comparative study was conducted. Pavón-Domínguez et al., (2014) estimated short-time ozone in Sevilla city (Spain) using a combination of hierarchical cluster analysis and multiple linear regressions. They obtained Pearson’s coefficients of correlation and RMSE values ranging from 0.911 to 0.943 and from 11.22 to 11.87 µg/m^3^, respectively. Similarly, Jung et al., (2024) developed a complex model (based on machine learning techniques) for estimating daily maximum 8-h average O_3_ levels. They conducted a cross-validation, obtaining a value of 0.906 (Pearson’s coefficient of correlation) and 15.37 µg/m^3^ (RMSE value. Expressed to 20 °C and 1013 hPa). Note that similar *r* values to the current study and RMSE values are significantly higher than those of this study. To simplify the outcomes obtained by other investigation groups, Table 3 is shown. The values of the statistical indicators obtained in the present study were 1.05 μg/m^3^ for RMSE, 1.11 μg/m^3^ for MAE, and 11.16% for MAPE. Note that the current study shows better performance in terms of absolute values and is more similar in terms of relative values. Nevertheless, this comparison is limited because most of the methods reported in Table 3 to estimate urban O_3_ concentrations differ from the current study, making it notably difficult to compare them. A possible explanation for the performance of the proposed approach is that it led to a larger number of independent variables (24 inputs) used to estimate the target air pollutant. Also, the collinearity between O_3_ and the input variables may favor estimating O_3_ relative to other cities. This aspect is notable to consider, as the presence of precursor O_3_ pollutants depends on topography, emission sources, and meteorological conditions, which differ among geographical zones. Note that reanalyzed pollution and meteorological data were used to develop the suggested mathematical model, so a possible overestimation of the performance could have occurred.

**Table 3:** Performance outcomes of notorious research groups in estimating urban O_3_ levels

City/country	Studied period	Methods	*r*^2^	RMSE (µg/m^3^)	MAE (µg/m^3^)	MAPE (%)	References
Madrid/Spain	2010–2018	Artificial neural networks (ANNs) + evolutionary optimization	~ 0.80–0.92	No calculated	No calculated	No calculated	Santos et al., (2021)
Several cities/China	2013–2017	Machine learning and physical model	~ 0.85–0.95	⁓9.97–19.94	⁓7.98–15.95	⁓10–25	Feng et al., (2019)
Several cities/Europe	2008–2010	Coupled meteorology-chemistry model	~ 0.60–0.80	⁓15.95–35.89	⁓11.96–27.91	No calculated	Mar et al., (2016)
Porto/Portugal	2000s	Linear statistical models	~ 0.70–0.85	⁓15.95–29.91	⁓11.96–19.94	No calculated	Pires et al., (2012)
Córdoba/Spain	1990s–2000s	Seasonal ARIMA time series models	~ 0.60–0.80	⁓19.94–39.88	No calculated	No calculated	Dueñas et al., (2005)
Porto/Portugal	2000s	Linear regression, time series, and ANNs	~ 0.75–0.90	⁓13.96–23.93	⁓9.97–17.95	No calculated	Sousa et al., (2006)
Several cities/EEUU	2001–2003	Deterministic chemical-transport model	~ 0.50–0.75	⁓19.94–49.85	⁓15.95–39.88	⁓20–35	Tong and Mauzerall, (2006)

The concentrations in µg/m^3^ are expressed to 20 °C and 1013 hPa

Given the scientific evidence obtained in the current study, the linear dependence of the dependent variable on the predictor variables is statistically significant, indicating the applicability of the proposed O_3_ estimation model in urban ambient air. This body of evidence supports the trustworthiness of the proposed mathematical expression for estimating urban daily O_3_ concentrations, regardless of time, which is essential for the soundness and plausibility of the target estimation model.

Over the covered time, air quality strategies have been developed at the European level and implemented nationally. Changes in air quality ultimately affect population exposure. The European Air Quality directives set air quality objectives to protect human beings’ health. These changes have been translated into reduced levels of primary air pollutants. Nevertheless, these control strategies have not been associated with declining ambient air O_3_ levels (see Fig. 1), further underscoring the need to develop estimation models for ambient air O_3_, with particular emphasis on urban areas due to elevated population density (Ravindiran et al., 2023).

As another innovative feature within the validation process, the proposed mathematical model was tested with respect to geography, since its applicability should be independent of the target urban environment. For that reason, the suggested methodological approach was implemented in a new urban environment. In particular, Bucharest (Romania) was selected as a significant European capital to test the validity of the geographical model. Bucharest is in eastern Europe (44° 25′ 56.1″ N, 26° 6′ 22.54″ E, according to https://latitudelongitude.org/). It is depicted by snow as a main climate, with fully humid precipitation and warm summer temperature (code: Dfb) according to the World Map of Köppen − Geiger Climate Classification (Kottek et al., 2006). In order to compare pollution with Madrid, Stockholm, and Rome in the time range utilized to develop the mathematical model, a 2004–2021 average concentration of 47.41 µg O_3_/m^3^ (95% CI 46.95–47.87 µg O_3_/m^3^, standard deviation 18.89 µg O_3_/m^3^, range 4.54–97.06 µg O_3_/m^3^) was observed in Bucharest. Note that this polluting scenery differs notably from that exhibited in Madrid, Stockholm, and Rome over the same period, which justified the selection of this major European city for testing the performance of the proposed estimating approach. Despite that, Bucharest qualitatively shares a similar seasonal pattern with Madrid and Rome, reaching the highest O_3_ levels in the warm period and warm vs. cold period differences of 27.57% (see Fig. S4A). The highest and lowest O_3_ levels fell in Summer (34.09% contribution) and Winter (14.84%) (Fig. S4B). Like Madrid, Stockholm, and Rome, the highest and lowest temperatures in Bucharest were Summer (296.38 K) and Winter (273.88 K), respectively, cooccurring with the maximum and minimum O_3_ concentrations (Fig. S4C).

In terms of weather, the most dominant variable regarding the O_3_ panorama in Bucharest was Pre unlike the other involved European cities, thereby sustaining another significant difference. The expression that relates to O_3_ levels in Bucharest and weather variables is O_3_ = 0.2419 T − 0.0041 × WD + 0.3027 × WS − 0.0825 × RH − 0.0714 × P + 0.5845 × Pre + 0.4486 × PBLH + 0.3065 × DRad + 0.0135. Regarding polluting compounds, Table S8 presents the outcomes of the correlation analysis between the dependent variable (O_3_) and each air pollutant predictor in Bucharest. Note that the prevailing compounds are similar to those in the previous three cities (Madrid, Stockholm, and Rome), except for airborne particulate matter (PM_10_ and PM_2.5_), thereby identifying a new difference among the covered cities. Considering 24 input variables, Table S9 reports the results of applying PCA-MLR analysis in Bucharest. A mean–variance value of 3.39% separated the dominant and non-dominant independent variables. Among the dominant variables, NO_3_^−^, CO, propane, and wind speed are shared with Madrid, Stockholm, and Rome; nevertheless, Bucharest have also as dominant predictor variables NO_2_, NO, SO_2_, PM_10_ and PM_2.5_, ethane, relative humidity, and PBLH, thereby sustaining notable differences concerning the rest of the studied European cities, which highlights the performance of the generated estimating model.

**Fig. 4:**
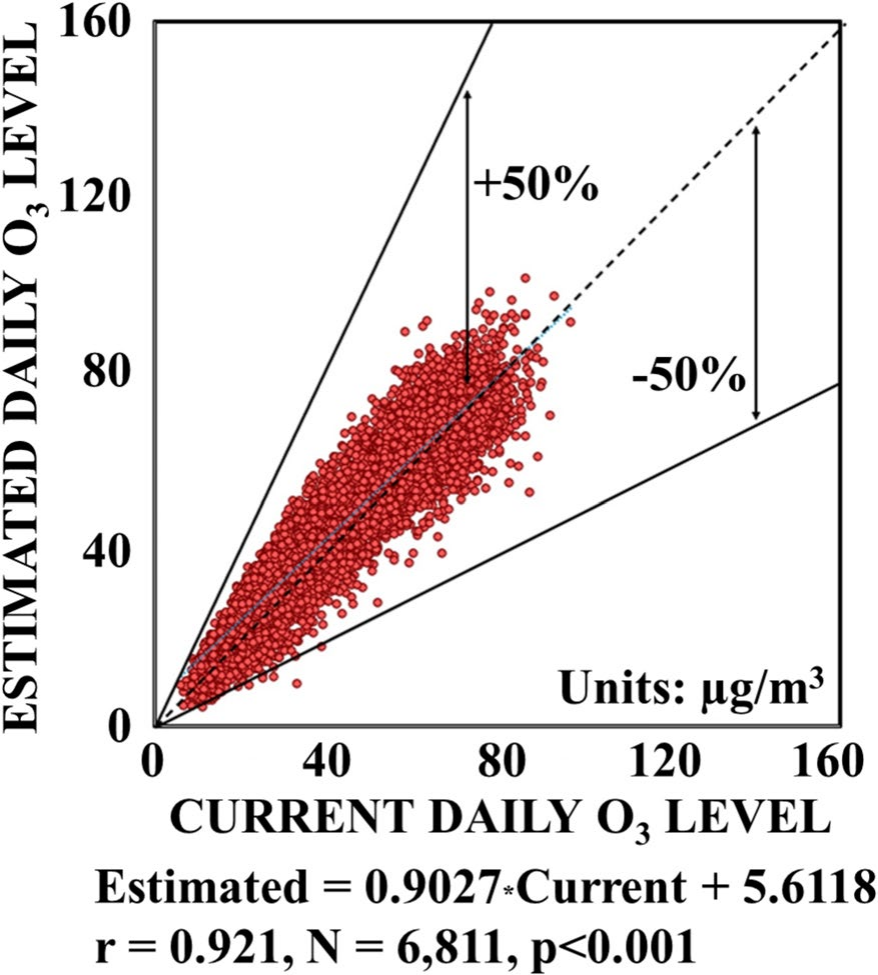
Scatterplot from 2004 to 2021 estimated vs. current daily O_3_ concentrations to test the geographical applicability of the proposed approach in Bucharest

The quantitative differences in pollution status and meteorological conditions between Bucharest and the selected cluster of cities used to develop the mathematical model, which lend significant value to selecting Bucharest for testing the model’s applicability in another urban environment. In this sense, Fig. 4 shows the linear regression model obtained when applying the proposed mathematical approach in Bucharest between 2004 and 2021. Note that a strong relationship between estimated and current daily O_3_ concentrations is obtained (*r* = 0.921), which implies that the vast set of input variables explains 85% of the total variance of the dependent variable. In terms of legislative, 98.8% of the data falls within the ± 50% legislative interval. In addition, the residues’ independence is secured (1.6). Regarding additional metric indicators, values of 1.79 µg/m^3^, 3.20 µg/m^3^, and 12.80% were obtained for RSME, MAE, and MAPE, respectively, and are reasonably similar to those reported for the set of cities used to develop the mathematical model, thereby allowing applying a similar explanation for these outcomes of performance indicators.

## Conclusion

Urban ozone pollution is a significant current environmental issue affecting urban sustainable development and public health. For this reason, this research aims to develop a mathematical expression that estimates daily urban ozone levels from satellite pollution and meteorological data. In this context, three major European cities (Madrid, Stockholm, and Rome) were chosen as a case study framework, thereby involving urban locations with distinct latitudes and longitudes, as well as morphology, emission sources, and meteorological features particular to each selected urban environment.

Unlike other published works within the suggested objective, this research study conducted a robust validation process to test the performance of the proposed approach, encompassing its application at both temporal and geographical scales as innovative aspects. The body of evidence supports the reliability and validity of the proposed approach for generating a standard mathematical expression that estimates daily ozone concentrations in urban environments using satellite pollution and meteorological data. In addition, as another innovative aspect, the short-term modeling criteria set by the current European legislation for ozone were compared with outcomes from the recommended approach, thereby ensuring compliance with current regulations. As limitations of the current study, reanalysis datasets (from satellite data) were used instead of independent observational data to engineer the proposed mathematical model, which may introduce uncertainties associated with the proposed approach and a lack of spatial representativeness.

In terms of practice application, the proposed ozone estimation model may be applied to other urban environments with existing satellite data for the set of predictor variables used in the present work. In this context, this approach may serve as a valuable tool to (i) complement the guidelines set by European legislation on ozone monitoring in urban areas in the function of modeling, (ii) identify urban locations with worrying exposure levels, (iii) ease in the decision making to urban environmental managers regarding the implementation of control tropospheric ozone strategies in potentially polluted areas.

In this sense, the ability of the proposed estimation model to reliably and accurately forecast urban ozone levels may provide additional, significant data on exposure and the spatial–temporal behavior derived from diverse polluting ozone sceneries, which is highly pertinent in the public health frame.

## Supplementary information

Below is the link to the electronic supplementary material.

ESM 1 (DOCX 1.18 MB)

## Data Availability

Data is available on request from the authors.
