# Supplementary material for: Estimating daily ozone levels in urban ambient air
Source: Environ Monit Assess. 2026 Jun 19;198(7):747. doi: 10.1007/s10661-026-15566-w (PMC13279484; doi:10.1007/s10661-026-15566-w)
Supplement: Supplementary file 1 — (DOCX 1.18 MB) [file 10661_2026_15566_MOESM1_ESM.docx]

**SUPPLEMENTARY DATA**

**Estimating daily ozone levels in urban ambient air**

David Galán-Madruga^1,2^*, Jafet Cárdenas-Escudero^2,3§^, Parya Broomandi^4^, J.L. Urraca^2^, Jorge O. Cáceres^2^

^1^National Reference Laboratory of Air Quality. National Centre for Environmental Health (CNSA). Carlos III Health Institute (ISCIII), Ctra. Majadahonda a Pozuelo, Madrid 28222, Spain

^2^Laser Chemistry Research Group, Department of Analytical Chemistry, Faculty of Chemistry, Complutense University of Madrid, Plaza de Ciencias 1, 28040 Madrid, Spain

^3^Analytical Chemistry Department, FCNET, University of Panama, University City, University Mail, 3366, Panama 4, Panama City, Panama

^4^Department of Civil and Environmental Engineering, School of Engineering and Digital Sciences, Nazarbayev University, Nursultan (Astana), Kazakhstan

^§^Doctoral Fellow, FCNET Universidad de Panamá

*Corresponding author: Dr. David Galan Madruga

Telephone: +34 918223507, fax: +34 915097991

e-mail address: [david.galan@isciii.es](mailto:david.galan@isciii.es)

David Galán-Madruga: ORCID: 0000-0002-7890-4611

**Tables**

**Table S1**. Outcomes reached when implementing the PCA technique in Madrid city.

**Table S2**. Outcomes reached when implementing the PCA technique in Stockholm city.

**Table S3**. Outcomes reached when implementing the PCA technique in Rome city.

**Table S4**. Cumulate variances when applying PCA technique for each covered city.

**Table S5**. Results reached when combining PCA-MLR technique in Madrid city.

**Table S6**. Results reached when combining PCA-MLR technique in Stockholm city.

**Table S7**. Results reached when combining PCA-MLR technique in Rome city.

**Table S8**. Outcomes obtained when applying correlation analysis between the dependent variable (O_3_) and each predictor input in Bucharest.

**Table S9**. Results reached when combining PCA-MLR technique in Bucharest.

**Figures**

**Fig. S1**. Warm vs. Cold 2004-2021 percentage distribution of ozone for each researched city. Note that the warm period involves Spring and Summer, while the cold period covers Autumn and Winter.

**Fig. S2**. Seasonal ozone distribution percentage for each researched city during the study period. Note that Spring includes March, April, and May; Summer involves June, July, and August; Autumn covers September, October, and November; and Winter includes December, January, and February.

**Fig. S3**. Monthly levels of ozone (µg/m^3^) and temperature (K) from 2004 to 2021 in Madrid, Stockholm, and Rome.

**Fig. S4**. A Graphic. Warm vs. Cold 2004-2021 percentage distribution of ozone in Bucharest (Note that the warm period involves Spring and Summer, while the cold period covers Autumn and Winter), B Graphic. Seasonal ozone percentage distribution in Bucharest (period: 2004-2021), and C: Monthly levels of ozone (µg/m^3^) and temperature (K) from 2004 to 2021 in Bucharest.

**TABLES**

**Table S1**. Outcomes reached when implementing the PCA technique in Madrid city.

| **Predictor variable** | **PC1** | **PC2** | **PC3** | **PC4** | **PC5** | **PC6** | **PC7** | **PC8** |
| --- | --- | --- | --- | --- | --- | --- | --- | --- |
| NO_2_ | 0.029 | -0.042 | **0.621** | 0.052 | -0.017 | 0.117 | -0.035 | 0.023 |
| NO | -0.264 | **0.652** | -0.103 | 0.474 | 0.063 | -0.166 | 0.031 | -0.021 |
| NO_3_^-^ | -0.095 | 0.030 | 0.002 | 0.015 | -0.012 | 0.014 | **0.991** | -0.005 |
| Peroxyacetyl nitrate | **0.843** | -0.090 | 0.035 | -0.069 | -0.024 | 0.080 | -0.017 | -0.032 |
| CO | -0.301 | **0.834** | -0.163 | 0.290 | 0.024 | -0.166 | 0.048 | 0.005 |
| SO_2_ | 0.025 | 0.508 | **-0.675** | 0.405 | 0.070 | -0.044 | 0.008 | -0.008 |
| PM_10_ | 0.035 | **0.895** | -0.161 | 0.051 | -0.046 | 0.153 | -0.029 | 0.018 |
| PM_2.5_ | 0.058 | **0.889** | -0.156 | 0.072 | -0.050 | 0.158 | -0.031 | 0.018 |
| PM_1_ | -0.034 | -0.195 | **0.781** | -0.005 | 0.073 | -0.050 | 0.021 | -0.010 |
| Methane | **-0.882** | 0.187 | 0.314 | 0.100 | -0.082 | -0.059 | 0.060 | -0.018 |
| Acetone | -0.056 | -0.162 | **0.837** | 0.044 | 0.016 | 0.066 | 0.030 | 0.003 |
| Ethane | -0.270 | 0.832 | -0.074 | -0.025 | -0.011 | -0.176 | 0.050 | 0.026 |
| Formaldehyde | **0.949** | 0.069 | 0.013 | 0.069 | 0.098 | -0.012 | -0.009 | 0.032 |
| Propane | -0.381 | 0.765 | -0.245 | 0.338 | 0.032 | -0.087 | 0.047 | -0.008 |
| Methanol | **0.912** | 0.057 | -0.025 | 0.143 | 0.146 | -0.018 | -0.011 | 0.014 |
| Methyl peroxy radicals | **0.926** | -0.182 | 0.106 | -0.013 | 0.007 | -0.058 | -0.017 | 0.016 |
| T | **0.921** | -0.238 | -0.013 | 0.031 | 0.016 | 0.013 | -0.052 | 0.027 |
| WD | 0.094 | -0.003 | 0.054 | -0.059 | **0.945** | 0.047 | -0.006 | 0.018 |
| WS | -0.235 | -0.217 | -0.015 | **-0.836** | -0.007 | -0.005 | 0.017 | 0.011 |
| RH | 0.040 | 0.033 | 0.023 | -0.008 | 0.016 | -0.004 | -0.005 | **0.997** |
| P | -0.096 | 0.153 | 0.097 | **0.610** | -0.396 | -0.334 | 0.047 | 0.013 |
| Pre | -0.132 | -0.028 | 0.178 | -0.098 | 0.064 | **0.883** | 0.018 | -0.003 |
| PBLH | **0.627** | -0.316 | 0.005 | -0.605 | -0.040 | -0.104 | -0.018 | 0.001 |
| DRad | **0.853** | -0.165 | -0.002 | -0.065 | -0.110 | -0.203 | -0.040 | 0.008 |
| Note: Higher factor loadings are marked in bold for each predictor variable. | | | | | | | | |

**Table S2**. Outcomes reached when implementing the PCA technique in Stockholm city.

| **Predictor variable** | **PC1** | **PC2** | **PC3** | **PC4** | **PC5** | **PC6** | **PC7** | **PC8** | **PC9** |
| --- | --- | --- | --- | --- | --- | --- | --- | --- | --- |
| NO_2_ | **0.565** | -0.318 | 0.176 | 0.117 | 0.472 | 0.182 | -0.063 | 0.134 | -0.018 |
| NO | -0.187 | -0.072 | -0.105 | 0.417 | **-0.584** | 0.089 | -0.056 | 0.160 | -0.079 |
| NO_3_^-^ | -0.081 | 0.010 | -0.003 | 0.011 | -0.022 | 0.032 | -0.011 | 0.007 | **0.989** |
| Peroxyacetyl nitrate | **0.824** | 0.260 | 0.047 | 0.103 | -0.068 | -0.020 | 0.015 | -0.079 | 0.030 |
| CO | -0.449 | **0.597** | -0.265 | 0.383 | -0.320 | 0.089 | 0.015 | 0.042 | 0.020 |
| SO_2_ | 0.109 | 0.230 | **-0.671** | 0.357 | -0.165 | 0.082 | 0.062 | 0.011 | 0.003 |
| PM_10_ | 0.141 | 0.102 | -0.165 | **0.933** | -0.041 | -0.031 | 0.079 | -0.041 | 0.012 |
| PM_2.5_ | 0.170 | 0.119 | -0.169 | **0.922** | -0.096 | -0.015 | 0.072 | -0.041 | 0.014 |
| PM_1_ | -0.015 | -0.110 | **0.831** | -0.075 | -0.018 | 0.041 | 0.042 | -0.036 | -0.024 |
| Methane | **-0.726** | 0.409 | 0.340 | -0.060 | -0.115 | 0.287 | -0.132 | -0.010 | 0.077 |
| Acetone | -0.002 | -0.018 | **0.849** | -0.101 | 0.019 | -0.082 | 0.009 | 0.037 | 0.021 |
| Ethane | -0.352 | **0.729** | -0.209 | 0.332 | -0.098 | 0.130 | -0.060 | 0.100 | 0.004 |
| Formaldehyde | **0.947** | -0.109 | -0.047 | 0.022 | -0.110 | -0.005 | 0.065 | -0.027 | -0.014 |
| Propane | -0.378 | **0.792** | -0.140 | 0.017 | -0.138 | -0.017 | 0.078 | -0.021 | -0.003 |
| Methanol | **0.912** | -0.098 | -0.051 | 0.038 | -0.211 | -0.006 | 0.005 | -0.022 | -0.011 |
| Methyl peroxy radicals | **0.863** | -0.308 | 0.059 | -0.030 | 0.035 | 0.028 | 0.004 | 0.040 | -0.032 |
| T | **0.816** | -0.407 | -0.062 | 0.051 | 0.074 | -0.074 | 0.127 | 0.034 | -0.062 |
| WD | 0.088 | 0.021 | 0.013 | 0.117 | 0.005 | -0.037 | **0.963** | 0.007 | -0.011 |
| WS | -0.307 | -0.010 | 0.005 | -0.021 | **0.803** | -0.136 | 0.079 | 0.003 | -0.022 |
| RH | 0.048 | 0.033 | -0.002 | -0.044 | 0.035 | 0.015 | 0.008 | **0.966** | 0.008 |
| P | 0.099 | 0.149 | 0.143 | 0.042 | -0.219 | **0.764** | -0.130 | 0.035 | 0.071 |
| Pre | 0.085 | 0.066 | 0.410 | 0.072 | -0.004 | **-0.720** | -0.106 | 0.018 | 0.039 |
| PBLH | -0.013 | -0.328 | 0.001 | -0.051 | **0.804** | -0.072 | -0.098 | 0.121 | -0.054 |
| DRad | **0.768** | -0.288 | -0.010 | 0.182 | 0.050 | 0.260 | -0.089 | 0.169 | -0.045 |
| Note: Higher factor loading is marked in bold for each predictor variable | | | | | | | | |  |

**Table S3**. Outcomes reached when implementing the PCA technique in Rome city.

| **Predictor variable** | **PC1** | **PC2** | **PC3** | **PC4** | **PC5** | **PC6** | **PC7** |
| --- | --- | --- | --- | --- | --- | --- | --- |
| NO_2_ | **0.783** | -0.335 | -0.384 | 0.026 | 0.070 | 0.008 | -0.013 |
| NO | -0.250 | **0.649** | 0.479 | -0.121 | -0.117 | 0.062 | 0.035 |
| NO_3_^-^ | -0.068 | 0.025 | 0.021 | 0.012 | 0.011 | 0.000 | **0.994** |
| Peroxyacetyl nitrate | **0.898** | -0.155 | 0.018 | -0.006 | -0.068 | -0.038 | 0.003 |
| CO | -0.374 | **0.676** | 0.477 | -0.257 | -0.156 | 0.023 | 0.046 |
| SO_2_ | 0.093 | 0.463 | 0.195 | **-0.779** | 0.050 | 0.008 | -0.003 |
| PM_10_ | -0.117 | **0.920** | 0.016 | -0.163 | 0.019 | -0.034 | -0.005 |
| PM_2.5_ | -0.105 | **0.920** | 0.033 | -0.162 | 0.018 | -0.034 | -0.007 |
| PM_1_ | -0.008 | -0.213 | 0.061 | **0.825** | -0.044 | -0.012 | 0.008 |
| Methane | **-0.845** | 0.100 | 0.241 | 0.280 | -0.201 | -0.016 | 0.063 |
| Acetone | -0.036 | -0.171 | 0.065 | **0.838** | 0.000 | 0.021 | 0.007 |
| Ethane | -0.428 | **0.646** | 0.317 | -0.368 | -0.145 | 0.012 | 0.045 |
| Formaldehyde | **0.944** | -0.063 | 0.110 | -0.001 | 0.018 | 0.033 | -0.013 |
| Propane | -0.342 | **0.675** | 0.457 | -0.368 | -0.065 | 0.020 | 0.027 |
| Methanol | **0.927** | -0.035 | 0.145 | -0.022 | 0.007 | 0.015 | -0.012 |
| Methyl peroxy radicals | **0.920** | -0.173 | -0.026 | 0.078 | 0.009 | 0.045 | -0.011 |
| T | **0.904** | -0.171 | -0.074 | 0.018 | 0.184 | 0.049 | -0.055 |
| WD | 0.096 | -0.103 | -0.083 | -0.048 | **0.902** | 0.000 | 0.013 |
| WS | -0.345 | -0.163 | **-0.716** | -0.032 | -0.099 | 0.142 | 0.035 |
| RH | 0.082 | -0.009 | -0.027 | 0.000 | -0.003 | **0.974** | -0.001 |
| P | -0.017 | 0.132 | **0.720** | 0.017 | -0.202 | 0.120 | 0.049 |
| Pre | -0.140 | 0.172 | **-0.447** | 0.440 | 0.348 | -0.030 | -0.010 |
| PBLH | 0.403 | -0.294 | **-0.684** | -0.052 | -0.108 | 0.153 | 0.017 |
| DRad | **0.862** | -0.121 | -0.001 | -0.040 | -0.104 | 0.032 | -0.010 |

Note: Higher factor loading is marked in bold for each predictor variable

**Table S4**. Cumulate variances when applying PCA technique in Madrid, Stockholm, and Rome.

| **Principal Component (PC)** | **Madrid** | **Stockholm** | **Rome** |
| --- | --- | --- | --- |
| PC1 | 33.05% | 28.96% | 37.50% |
| PC2 | 19.89% | 17.41% | 19.16% |
| PC3 | 8.18% | 8.59% | 8.87% |
| PC4 | 6.37% | 6.46% | 5.31% |
| PC5 | 4.27% | 5.44% | 4.42% |
| PC6 | 4.10% | 4.17% | 4.12% |
| PC7 | 4.05% | 4.05% | 3.63% |
| PC8 | 3.47% | 3.88% | --- |
| PC9 | --- | 3.67% | --- |
| Cumulate variance | 83.38% | 82.62% | 83.00% |

**Table S5**. Results reached when combining PCA-MLR technique in Madrid city.

| **Predictor variable** | **PC1** | **PC2** | **PC3** | **PC4** | **PC5** | **PC6** | **PC7** | **PC8** | **Variance**  **Total** |
| --- | --- | --- | --- | --- | --- | --- | --- | --- | --- |
| NO_2_ | 0.00% | 0.01% | 1.28% | 0.01% | 0.00% | 0.05% | 0.00% | 0.00% | 1.36% |
| NO | 0.35% | 1.82% | 0.04% | 0.68% | 0.01% | 0.10% | 0.00% | 0.00% | 3.01% |
| NO_3_^-^ | 0.05% | 0.00% | 0.00% | 0.00% | 0.00% | 0.00% | 3.95% | 0.00% | 4.00% |
| Peroxyacetyl nitrate | 3.59% | 0.03% | 0.00% | 0.01% | 0.00% | 0.02% | 0.00% | 0.00% | 3.67% |
| CO | 0.46% | 2.97% | 0.09% | 0.26% | 0.00% | 0.10% | 0.01% | 0.00% | 3.88% |
| SO_2_ | 0.00% | 1.10% | 1.51% | 0.50% | 0.02% | 0.01% | 0.00% | 0.00% | 3.14% |
| PM_10_ | 0.01% | 3.42% | 0.09% | 0.01% | 0.01% | 0.09% | 0.00% | 0.00% | 3.62% |
| PM_2.5_ | 0.02% | 3.38% | 0.08% | 0.02% | 0.01% | 0.09% | 0.00% | 0.00% | 3.60% |
| PM_1_ | 0.01% | 0.16% | 2.03% | 0.00% | 0.02% | 0.01% | 0.00% | 0.00% | 2.23% |
| Methane | 3.93% | 0.15% | 0.33% | 0.03% | 0.03% | 0.01% | 0.01% | 0.00% | 4.49% |
| Acetone | 0.02% | 0.11% | 2.33% | 0.01% | 0.00% | 0.02% | 0.00% | 0.00% | 2.49% |
| Ethane | 0.37% | 2.96% | 0.02% | 0.00% | 0.00% | 0.11% | 0.01% | 0.00% | 3.47% |
| Formaldehyde | 4.55% | 0.02% | 0.00% | 0.01% | 0.04% | 0.00% | 0.00% | 0.00% | 4.63% |
| Propane | 0.73% | 2.50% | 0.20% | 0.35% | 0.00% | 0.03% | 0.01% | 0.00% | 3.82% |
| Methanol | 4.20% | 0.01% | 0.00% | 0.06% | 0.08% | 0.00% | 0.00% | 0.00% | 4.36% |
| Methyl peroxy radicals | 4.34% | 0.14% | 0.04% | 0.00% | 0.00% | 0.01% | 0.00% | 0.00% | 4.53% |
| T | 4.29% | 0.24% | 0.00% | 0.00% | 0.00% | 0.00% | 0.01% | 0.00% | 4.55% |
| WD | 0.04% | 0.00% | 0.01% | 0.01% | 3.38% | 0.01% | 0.00% | 0.00% | 3.45% |
| WS | 0.28% | 0.20% | 0.00% | 2.13% | 0.00% | 0.00% | 0.00% | 0.00% | 2.61% |
| RH | 0.01% | 0.00% | 0.00% | 0.00% | 0.00% | 0.00% | 0.00% | 3.44% | 3.46% |
| P | 0.05% | 0.10% | 0.03% | 1.13% | 0.59% | 0.41% | 0.01% | 0.00% | 2.32% |
| Pre | 0.09% | 0.00% | 0.11% | 0.03% | 0.02% | 2.84% | 0.00% | 0.00% | 3.08% |
| PBLH | 1.99% | 0.43% | 0.00% | 1.11% | 0.01% | 0.04% | 0.00% | 0.00% | 3.58% |
| DRad | 3.68% | 0.12% | 0.00% | 0.01% | 0.05% | 0.15% | 0.01% | 0.00% | 4.01% |
| Variance | 33.05% | 19.89% | 8.18% | 6.37% | 4.27% | 4.10% | 4.05% | 3.47% | **83.38%** |

**Table S6**. Results reached when combining PCA-MLR technique in Stockholm city.

| **Predictor variable** | **PC1** | **PC2** | **PC3** | **PC4** | **PC5** | **PC6** | **PC7** | **PC8** | **PC9** | **Variance**  **Total** |
| --- | --- | --- | --- | --- | --- | --- | --- | --- | --- | --- |
| NO_2_ | 1.55% | 0.72% | 0.11% | 0.04% | 0.56% | 0.10% | 0.02% | 0.07% | 0.00% | 3.17% |
| NO | 0.17% | 0.04% | 0.04% | 0.47% | 0.86% | 0.02% | 0.01% | 0.09% | 0.02% | 1.73% |
| NO_3_^-^ | 0.03% | 0.00% | 0.00% | 0.00% | 0.00% | 0.00% | 0.00% | 0.00% | 3.55% | 3.59% |
| Peroxyacetyl nitrate | 3.31% | 0.48% | 0.01% | 0.03% | 0.01% | 0.00% | 0.00% | 0.02% | 0.00% | 3.87% |
| CO | 0.98% | 2.54% | 0.25% | 0.40% | 0.26% | 0.02% | 0.00% | 0.01% | 0.00% | 4.46% |
| SO_2_ | 0.06% | 0.38% | 1.60% | 0.35% | 0.07% | 0.02% | 0.01% | 0.00% | 0.00% | 2.49% |
| PM_10_ | 0.10% | 0.07% | 0.10% | 2.35% | 0.00% | 0.00% | 0.02% | 0.01% | 0.00% | 2.66% |
| PM_2.5_ | 0.14% | 0.10% | 0.10% | 2.30% | 0.02% | 0.00% | 0.02% | 0.01% | 0.00% | 2.69% |
| PM_1_ | 0.00% | 0.09% | 2.46% | 0.02% | 0.00% | 0.01% | 0.01% | 0.00% | 0.00% | 2.58% |
| Methane | 2.56% | 1.19% | 0.41% | 0.01% | 0.03% | 0.25% | 0.07% | 0.00% | 0.02% | 4.55% |
| Acetone | 0.00% | 0.00% | 2.56% | 0.03% | 0.00% | 0.02% | 0.00% | 0.01% | 0.00% | 2.62% |
| Ethane | 0.60% | 3.78% | 0.16% | 0.30% | 0.02% | 0.05% | 0.01% | 0.04% | 0.00% | 4.96% |
| Formaldehyde | 4.37% | 0.08% | 0.01% | 0.00% | 0.03% | 0.00% | 0.02% | 0.00% | 0.00% | 4.51% |
| Propane | 0.70% | 4.47% | 0.07% | 0.00% | 0.05% | 0.00% | 0.02% | 0.00% | 0.00% | 5.31% |
| Methanol | 4.05% | 0.07% | 0.01% | 0.00% | 0.11% | 0.00% | 0.00% | 0.00% | 0.00% | 4.25% |
| Methyl peroxy radicals | 3.62% | 0.67% | 0.01% | 0.00% | 0.00% | 0.00% | 0.00% | 0.01% | 0.00% | 4.33% |
| T | 3.24% | 1.18% | 0.01% | 0.01% | 0.01% | 0.02% | 0.06% | 0.00% | 0.01% | 4.55% |
| WD | 0.04% | 0.00% | 0.00% | 0.04% | 0.00% | 0.00% | 3.57% | 0.00% | 0.00% | 3.65% |
| WS | 0.46% | 0.00% | 0.00% | 0.00% | 1.62% | 0.06% | 0.02% | 0.00% | 0.00% | 2.17% |
| RH | 0.01% | 0.01% | 0.00% | 0.01% | 0.00% | 0.00% | 0.00% | 3.45% | 0.00% | 3.48% |
| P | 0.05% | 0.16% | 0.07% | 0.00% | 0.12% | 1.78% | 0.06% | 0.00% | 0.02% | 2.27% |
| Pre | 0.03% | 0.03% | 0.60% | 0.01% | 0.00% | 1.58% | 0.04% | 0.00% | 0.01% | 2.31% |
| PBLH | 0.00% | 0.76% | 0.00% | 0.01% | 1.63% | 0.02% | 0.04% | 0.05% | 0.01% | 2.52% |
| DRad | 2.87% | 0.59% | 0.00% | 0.09% | 0.01% | 0.21% | 0.03% | 0.11% | 0.01% | 0.04 |
| Variance | 28.96% | 17.41% | 8.59% | 6.46% | 5.44% | 4.17% | 4.05% | 3.88% | 3.67% | **82.62%** |

**Table S7**. Results reached when combining PCA-MLR technique in Rome city.

| **Predictor variable** | **PC1** | **PC2** | **PC3** | **PC4** | **PC5** | **PC6** | **PC7** | **Variance**  **Total** |
| --- | --- | --- | --- | --- | --- | --- | --- | --- |
| NO_2_ | 3.22% | 0.52% | 0.47% | 0.00% | 0.02% | 0.00% | 0.00% | 4.23% |
| NO | 0.33% | 1.96% | 0.74% | 0.03% | 0.05% | 0.02% | 0.00% | 3.12% |
| NO_3_^-^ | 0.02% | 0.00% | 0.00% | 0.00% | 0.00% | 0.00% | 3.56% | 3.59% |
| Peroxyacetyl nitrate | 4.23% | 0.11% | 0.00% | 0.00% | 0.02% | 0.01% | 0.00% | 4.36% |
| CO | 0.73% | 2.12% | 0.73% | 0.13% | 0.09% | 0.00% | 0.01% | 3.81% |
| SO_2_ | 0.05% | 1.00% | 0.12% | 1.20% | 0.01% | 0.00% | 0.00% | 2.37% |
| PM_10_ | 0.07% | 3.93% | 0.00% | 0.05% | 0.00% | 0.00% | 0.00% | 4.06% |
| PM_2.5_ | 0.06% | 3.93% | 0.00% | 0.05% | 0.00% | 0.00% | 0.00% | 4.04% |
| PM_1_ | 0.00% | 0.21% | 0.01% | 1.35% | 0.01% | 0.00% | 0.00% | 1.58% |
| Methane | 3.75% | 0.05% | 0.19% | 0.16% | 0.15% | 0.00% | 0.01% | 4.30% |
| Acetone | 0.01% | 0.14% | 0.01% | 1.39% | 0.00% | 0.00% | 0.00% | 1.55% |
| Ethane | 0.96% | 1.93% | 0.32% | 0.27% | 0.08% | 0.00% | 0.01% | 3.57% |
| Formaldehyde | 4.67% | 0.02% | 0.04% | 0.00% | 0.00% | 0.00% | 0.00% | 4.73% |
| Propane | 0.61% | 2.11% | 0.67% | 0.27% | 0.02% | 0.00% | 0.00% | 3.69% |
| Methanol | 4.51% | 0.01% | 0.07% | 0.00% | 0.00% | 0.00% | 0.00% | 4.58% |
| Methyl peroxy radicals | 4.44% | 0.14% | 0.00% | 0.01% | 0.00% | 0.01% | 0.00% | 4.61% |
| T | 4.28% | 0.14% | 0.02% | 0.00% | 0.13% | 0.01% | 0.01% | 4.59% |
| WD | 0.05% | 0.05% | 0.02% | 0.00% | 3.10% | 0.00% | 0.00% | 3.22% |
| WS | 0.62% | 0.12% | 1.64% | 0.00% | 0.04% | 0.08% | 0.00% | 2.52% |
| RH | 0.04% | 0.00% | 0.00% | 0.00% | 0.00% | 3.82% | 0.00% | 3.86% |
| P | 0.00% | 0.08% | 1.66% | 0.00% | 0.15% | 0.06% | 0.01% | 1.97% |
| Pre | 0.10% | 0.14% | 0.64% | 0.38% | 0.46% | 0.00% | 0.00% | 1.73% |
| PBLH | 0.85% | 0.40% | 1.50% | 0.01% | 0.04% | 0.09% | 0.00% | 2.90% |
| DRad | 3.90% | 0.07% | 0.00% | 0.00% | 0.04% | 0.00% | 0.00% | 4.02% |
| Variance | 37.50% | 19.16% | 8.87% | 5.31% | 4.42% | 4.12% | 3.63% | **83.00%** |

**Table S8**. Outcomes obtained when applying correlation analysis between the dependent variable (O_3_) and each predictor input in Bucharest.

|  | **Bucharest** | | |
| --- | --- | --- | --- |
| **Predictor input** | **r^a^** | **Lower CI^b^** | **Upper CI** |
| NO_2_ | **1.000** | **---** | **---** |
| NO | **-0.618** | -0.633 | -0.603 |
| Nitratos | -0.112 | -0.135 | -0.088 |
| Peroxyacetyl nitrate | 0.455 | 0.435 | 0.474 |
| CO | **-0.730** | -0.741 | -0.719 |
| SO_2_ | -0.468 | -0.486 | -0.449 |
| PM_10_ | **-0.503** | -0.521 | -0.485 |
| PM_2.5_ | **-0.503** | -0.520 | -0.484 |
| PM_1_ | 0.017 | -0.007 | 0.041 |
| Methane | **-0.678** | -0.690 | -0.664 |
| Acetone | 0.040 | 0.015 | 0.064 |
| Ethane | **-0.590** | -0.606 | -0.574 |
| Formaldehyde | 0.316 | 0.295 | 0.338 |
| Propane | **-0.726** | -0.738 | -0.715 |
| Methanol | **0.531** | 0.513 | 0.548 |
| Peroxy radicals | **0.714** | 0.702 | 0.726 |
| Key: ^a^ Pearson’s coefficient of correlation, and ^b^ Confidence interval | | | |
|  | | | |

**Table S9**. Results reached when combining PCA-MLR technique in Bucharest.

| **Predictor variable** | **PC1** | **PC2** | **PC3** | **PC4** | **PC5** | **PC6** | **PC7** | **Variance**  **Total** |
| --- | --- | --- | --- | --- | --- | --- | --- | --- |
| NO_2_ | 2.44% | 1.05% | 0.08% | 0.06% | 0.03% | 0.02% | 0.00% | 3.68% |
| NO | 4.23% | 0.05% | 0.02% | 0.00% | 0.04% | 0.00% | 0.00% | 4.34% |
| NO_3_^-^ | 0.01% | 0.03% | 0.00% | 0.00% | 0.00% | 0.00% | 3.93% | 3.96% |
| Peroxyacetyl nitrate | 0.04% | 1.31% | 0.17% | 0.00% | 0.08% | 0.02% | 0.02% | 1.64% |
| CO | 4.60% | 0.23% | 0.07% | 0.01% | 0.03% | 0.00% | 0.00% | 4.95% |
| SO_2_ | 2.83% | 0.00% | 1.09% | 0.10% | 0.03% | 0.00% | 0.00% | 4.05% |
| PM_10_ | 3.95% | 0.02% | 0.54% | 0.01% | 0.00% | 0.00% | 0.00% | 4.52% |
| PM_2.5_ | 3.96% | 0.02% | 0.54% | 0.01% | 0.00% | 0.00% | 0.00% | 4.53% |
| PM_1_ | 0.18% | 0.00% | 2.31% | 0.00% | 0.00% | 0.00% | 0.00% | 2.49% |
| Methane | 0.48% | 2.03% | 0.45% | 0.02% | 0.09% | 0.00% | 0.03% | 3.09% |
| Acetone | 0.16% | 0.00% | 2.38% | 0.00% | 0.03% | 0.00% | 0.00% | 2.58% |
| Ethane | 3.83% | 0.15% | 0.31% | 0.00% | 0.00% | 0.00% | 0.00% | 4.30% |
| Formaldehyde | 0.18% | 2.48% | 0.22% | 0.02% | 0.00% | 0.00% | 0.00% | 2.91% |
| Propane | 4.06% | 0.07% | 0.31% | 0.07% | 0.07% | 0.00% | 0.00% | 4.59% |
| Methanol | 0.02% | 2.70% | 0.00% | 0.00% | 0.00% | 0.00% | 0.00% | 2.72% |
| Methyl peroxy radicals | 0.44% | 2.44% | 0.00% | 0.01% | 0.00% | 0.01% | 0.01% | 2.91% |
| T | 0.69% | 2.24% | 0.06% | 0.00% | 0.01% | 0.00% | 0.03% | 3.03% |
| WD | 0.04% | 0.00% | 0.02% | 2.86% | 0.00% | 0.00% | 0.00% | 2.93% |
| WS | 1.19% | 0.40% | 0.11% | 0.77% | 0.10% | 0.03% | 0.01% | 2.60% |
| RH | 0.00% | 0.00% | 0.00% | 0.00% | 0.00% | 3.99% | 0.00% | 4.00% |
| P | 0.26% | 0.19% | 0.14% | 0.45% | 1.58% | 0.00% | 0.02% | 2.64% |
| Pre | 0.02% | 0.01% | 0.23% | 0.18% | 2.42% | 0.00% | 0.00% | 2.86% |
| PBLH | 2.31% | 0.47% | 0.26% | 0.29% | 0.00% | 0.07% | 0.00% | 3.41% |
| DRad | 0.42% | 2.07% | 0.08% | 0.01% | 0.08% | 0.02% | 0.01% | 2.69% |
| Variance | 36.34% | 17.97% | 9.36% | 4.88% | 4.62% | 4.17% | 4.07% | **81.41%** |

**FIGURES**


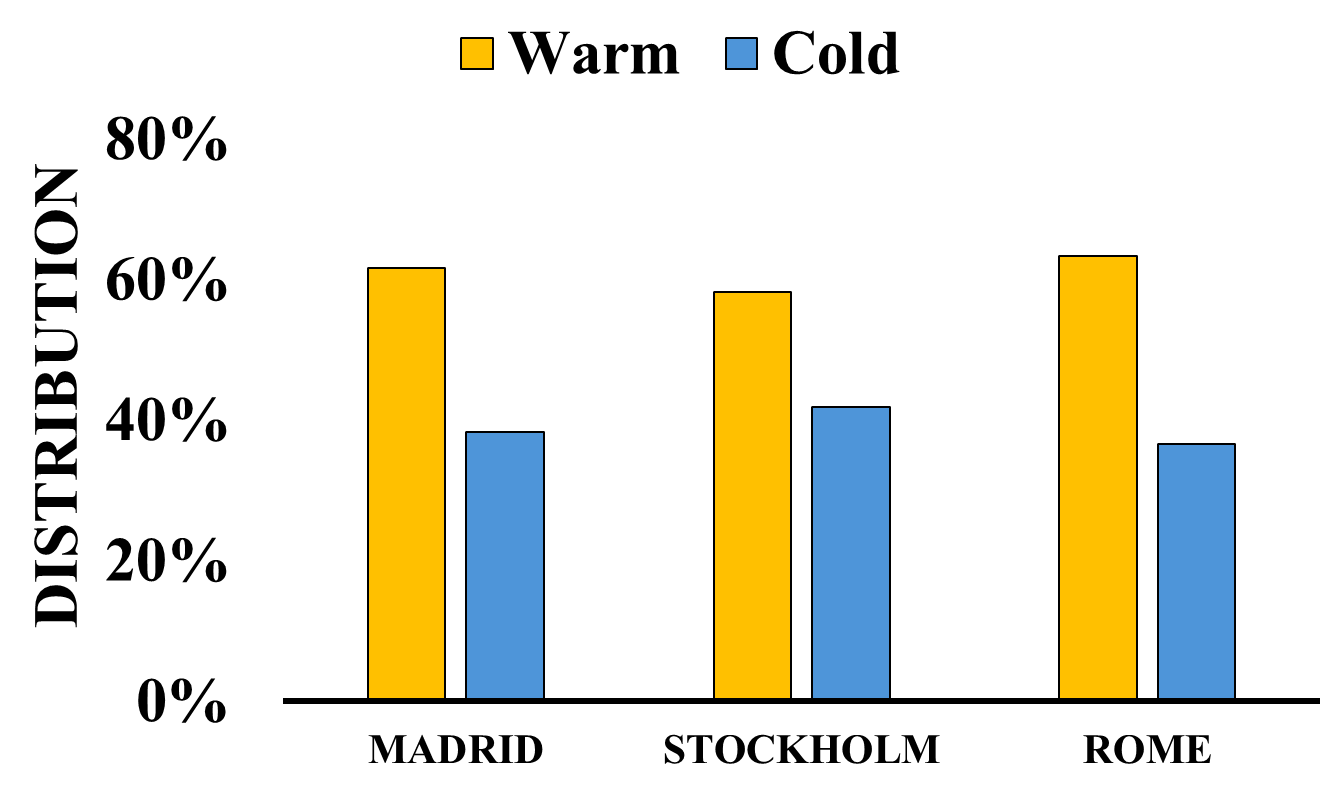


**Fig. S1**. Warm vs. Cold 2004-2021 percentage distribution of ozone for each researched city. Note that the warm period involves Spring and Summer, while the cold period covers Autumn and Winter.


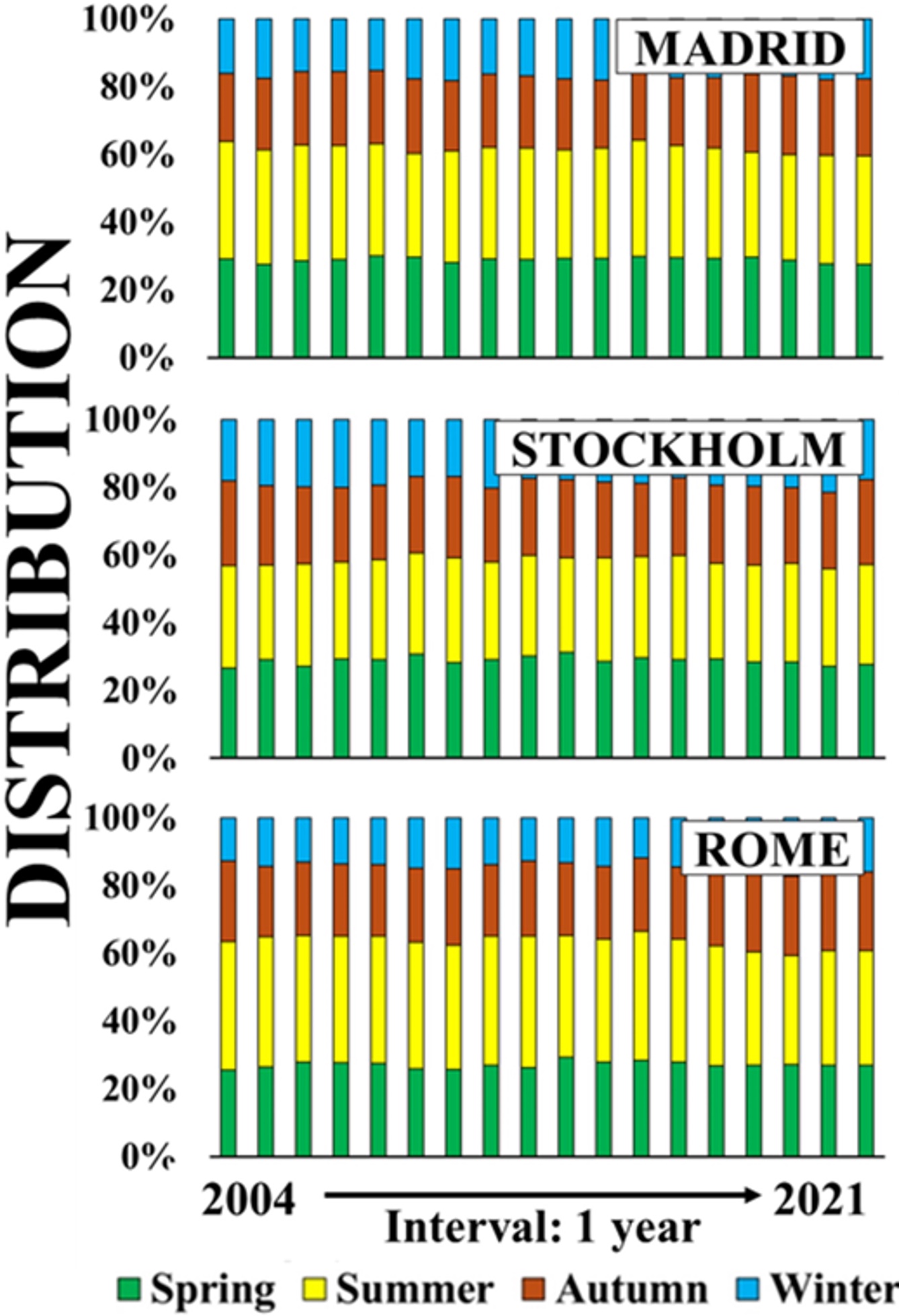


**Fig. S2**. Seasonal ozone distribution percentage for each researched city during the study period. Note that Spring includes March, April, and May; Summer involves June, July, and August; Autumn covers September, October, and November; and Winter includes December, January, and February.


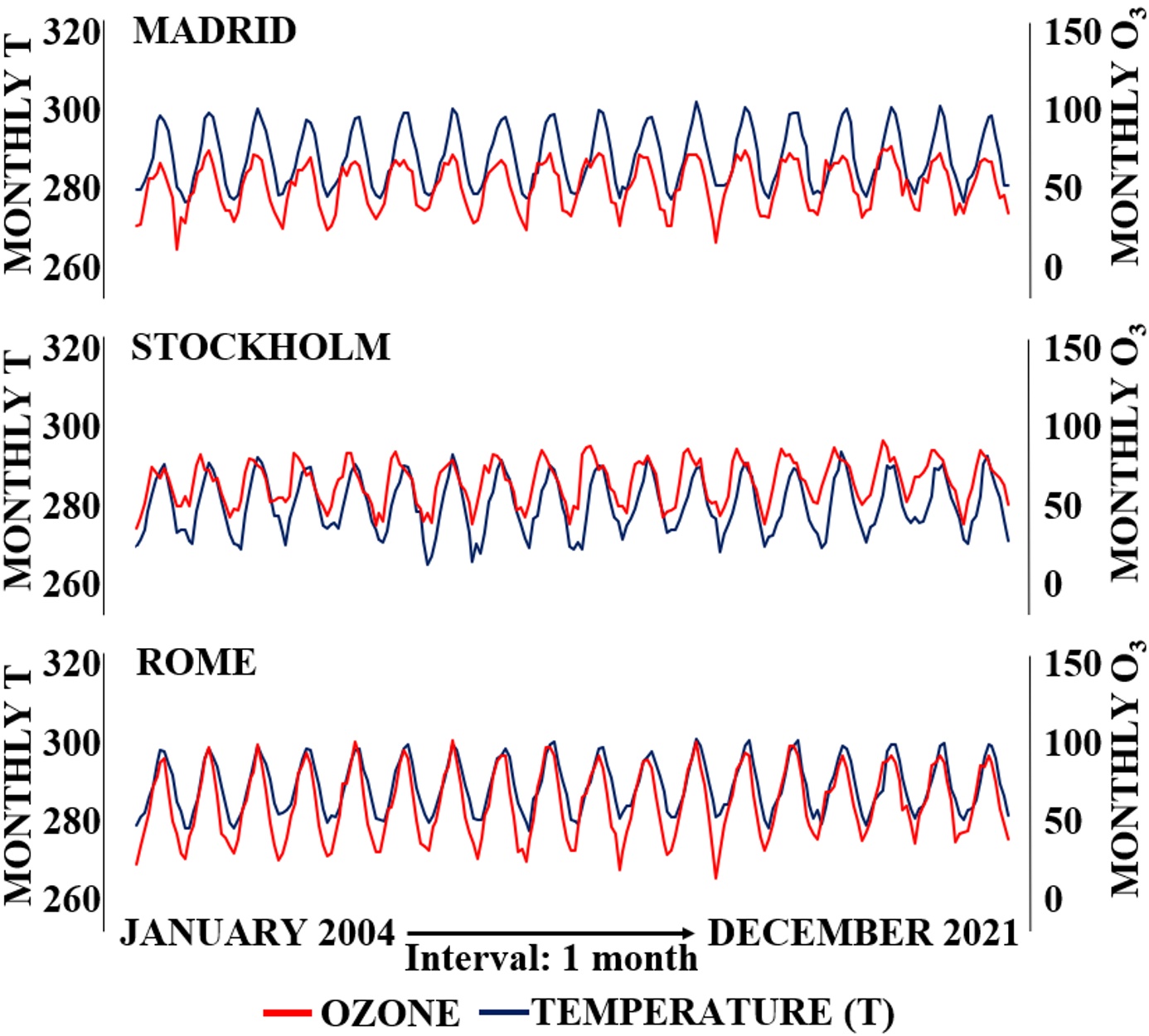


**Fig. S3**. Monthly levels of ozone (µg/m^3^) and temperature (K) from 2004 to 2021 in Madrid, Stockholm, and Rome.

**
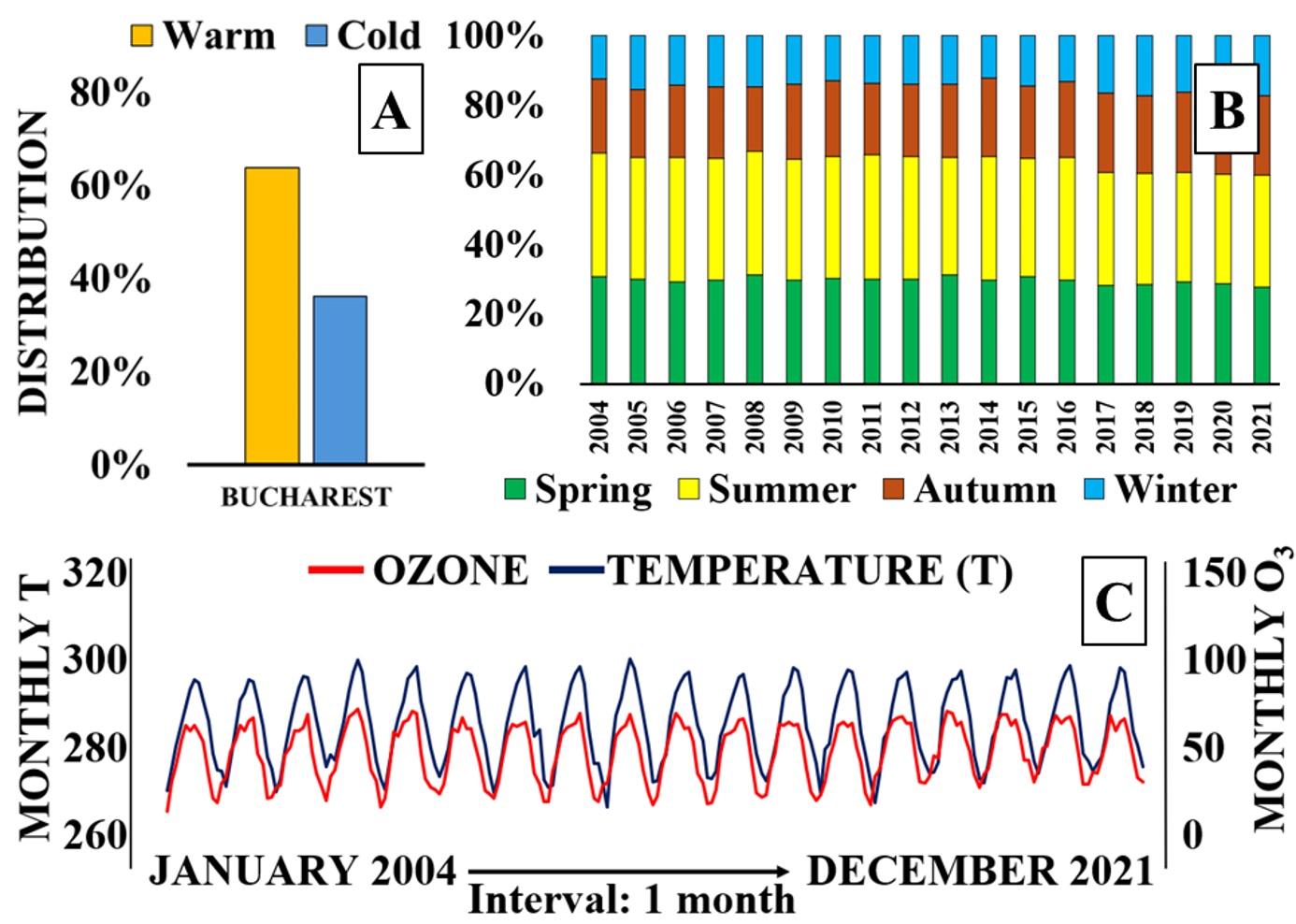
**

**Fig. S4**. A Graphic. Warm vs. Cold 2004-2021 percentage distribution of ozone in Bucharest (Note that the warm period involves Spring and Summer, while the cold period covers Autumn and Winter), B Graphic. Seasonal ozone percentage distribution in Bucharest (period: 2004-2021), and C: Monthly levels of ozone (µg/m^3^) and temperature (K) from 2004 to 2021 in Bucharest.
